# Infantile Krabbe disease (0–12 months), progression, and recommended endpoints for clinical trials

**DOI:** 10.1002/acn3.52114

**Published:** 2024-11-05

**Authors:** Melissa R. Greco, Mabel A. Lopez, Maria L. Beltran‐Quintero, Ecenur Tuc Bengur, Michele D. Poe, Maria L. Escolar

**Affiliations:** ^1^ Department of Genetics University of Pittsburgh Pittsburgh Pennsylvania USA; ^2^ Department of Pediatrics University of Pittsburgh Pittsburgh Pennsylvania USA; ^3^ Forge Biologics Grove City Ohio USA

## Abstract

**Objective:**

Krabbe disease is due to deficiency of galactocerebrosidase, resulting in progressive neurodegeneration due to demyelination. The purpose of this study is to document disease progression in the newly classified infantile‐onset (0–12 months). We evaluated the outcomes of hematopoietic stem cell transplantation (HSCT) and described meaningful clinical endpoints.

**Methods:**

Patients with infantile Krabbe disease were prospectively evaluated between 2000 and 2022. All patients underwent comprehensive and standardized protocols. Descriptive statistics and Kaplan–Meier survival curves were used for analysis.

**Results:**

One hundred and thirty‐seven children with infantile Krabbe disease were included (68 males and 69 females). Of the 137, 96 were not treated and 41 underwent hematopoietic stem cell transplantation. Twenty‐three were asymptomatic and 18 symptomatic. Initial symptoms included irritability, developmental delay or loss of milestones, feeding difficulties, spasticity, and reflux with an average survival of 2.2. Abnormalities in nerve conduction studies, auditory brainstem responses, and brain MRIs were evident in both groups of patients. Age at transplantation and signs and symptoms determined functional outcomes. Symptomatic and asymptomatic transplanted patients showed an increase in galactocerebrosidase and a decrease in psychosine, but did not reach the normal range. The median survival for transplanted symptomatic patients was 5 years while asymptomatic was extended to 15.5 years.

**Interpretation:**

Infantile Krabbe disease with onset before 12 months is rapidly progressive. Irreversible brain damage occurs unless timely HSCT is performed. HSCT does not prevent the progression of peripheral nerve disease. This study can be used to monitor patients and evaluate the effects of future therapies.

## Introduction

Krabbe disease (KD), or globoid cell leukodystrophy, is an autosomal recessive disorder caused by biallelic mutations in galactocerebrosidase (GALC).^1^, ^2^, ^3^ GALC is essential for the metabolism of galactosylceramide, a key component of myelin. A mutation in GALC results in the accumulation of psychosine that is extremely toxic to oligodendrocytes and Schwann cells, resulting in progressive demyelination of the nervous system.^4^, ^5^, ^6^, ^7^

The current standard of care for KD is hematopoietic stem cell transplantation (HSCT). HSCT introduces donor‐derived cells, which offer cross correction to GALC deficient oligodendrocytes in the brain. However, HSCT can stabilize disease only when symptoms are evident. Outcomes of symptomatic patients are similar to untreated patients, and complications related to HSCT are more likely to lead to death.^8^, ^9^, ^10^, ^11^

In 2006, newborn screening (NBS) for KD was introduced in New York, and other programs are being implemented throughout the United States.^12^, ^13^, ^14^ Currently, there are no reliable studies correlating genotype, GALC activity, psychosine levels, and clinical progression. Therefore, the timing of treatment decisions for children at risk of disease remains a significant challenge. Recently, psychosine levels in dried blood spots (DBS) have been used to predict disease progression in severe infantile KD patients. However, there is debate among experts regarding the cutoff to identify a patient as “at‐risk” for the infantile‐onset, and there are few studies correlating psychosine levels and later‐onset phenotypes.^15^ A detailed natural history and transplantation outcomes is necessary in patients with a disease onset between 0 and 12 months to monitor and recommend treatment in NBS‐positive patients in which psychosine levels cannot predict onset.

Historically, KD has been divided into four subtypes based on symptom onset: early infantile (0–5 months), late‐infantile (6–36 months), juvenile (37 months‐16 years), and adult (>16 years).^1^ The early infantile is the most common and aggressive form, affecting 80% of all patients with KD.^16^, ^17^ Clinical manifestations include feeding difficulties, irritability, psychomotor regression, spasticity, seizures, vision loss, hearing issues, and premature death.^18^, ^19^ Around 20% of patients with the late‐infantile form experience a slower progression and variable symptoms, such as vision loss, psychomotor regression, spastic paraparesis, and gait abnormalities.^20^, ^21^ Meanwhile, the juvenile and adult forms typically present as gait abnormalities and vision and hearing loss.^22^, ^23^

More recently, a detailed prospective and well‐standardized natural history study of KD with symptom onset from 6 to 36 months was published by Bascou et al. and subsequently, Beltran‐Quintero et al., published findings in those with symptom onset from birth to 6 months.^1^, ^18^ Both found that patients from 6 to 12 months had the same clinical presentation as those with onset from 0 to 6 months. Because this is the only prospective study with a large number of patients, the early infantile and the 6–12 months within late‐infantile were re‐classified as “infantile” to include patients with symptom onset from 0 to 12 months.

To date, there are no publications that combine the data from these natural history studies and that characterize these two populations combined. As a result, there remains a significant need to demonstrate the variability of progression in the 0–12‐month group using a large cohort. Given the differences in functional outcomes between asymptomatic and symptomatic transplanted patients, as reported in Yoon et al., it is imperative that the onset risk is well understood when recommending referral to transplant.^24^ With the emergence of new therapies, there is also a critical need to understand how they alter the progression of the infantile KD transplanted cohort. The purpose of this study is to longitudinally describe the neurodevelopmental manifestations of the 0–12‐month age group and to develop clinical endpoints to evaluate novel therapies.

## Methods

### Patient selection

Patients with disease onset between 0 and 12 months were evaluated between January 2000 and August 2022 at the Program for the Study of Neurodevelopment in Rare Disorders (NDRD) at the Children's Hospital of Pittsburgh and at the University of North Carolina. Diagnosis of Krabbe disease was determined based on GALC activity in fibroblasts or lysed leukocytes and confirmed by genetic analysis. Before 2009, diagnosis was based on GALC deficiency and/or family history or neuroimaging/neurophysiological studies. In addition, after 2009, diagnosis was made using genetic mutations predictive of abnormal function of GALC (Table S1). After 2017, psychosine was available clinically and added to the diagnostic algorithm, and patients with elevated psychosine (>2 nmol/L) were considered to have a positive psychosine test.

Patients referred to NDRD were offered enrollment into a longitudinal research study approved by the IRBs at the University of Pittsburgh (IRB‐PRO11050036) and the University of North Carolina (IRB‐08‐023). One hundred forty‐four patients were evaluated and 137 met the inclusion criteria for the prospective protocol. Seven patients were excluded because informed consent was not obtained. Patients were evaluated between 0.1 and 60 months.

### Neurodevelopmental testing

Subjects were assessed using a comprehensive standardized testing designed by a multidisciplinary team for longitudinal follow‐up.^25^ Neurodevelopmental pediatricians, neurophysiologists, physical therapists, speech pathologists, audiologists, and psychometricians evaluated patients for 4–6 h. Assessments included physical and neurological examinations, which assessed the signs and symptoms of disease, physical characteristics, growth, mobility, adaptive and cognitive behavior, sensory function, and speech and language skills. The Mullen Scales of Early Learning, Gross Motor Function Measure, The Peabody Developmental Motor Scales | Second Edition (PDMS‐II), and Vineland Adaptive Motor Scales were used to evaluate developmental function.^25^, ^26^, ^27^, ^28^, ^29^ For additional details about methodology, please see Supplementary Text and Table S2.

Parents completed a questionnaire that asked about birth history, early signs of disease, development, and behavior, such as the emergence of independent‐adaptive behavior. Patient outcomes were compared to the norms of typically developing children.^25^ Survival data were obtained by parent reports, internet searches, and/or querying the United States Social Security Death Index.

### Diagnostic testing

GALC activity in fibroblasts or lysed leukocytes was tested at the Lysosomal Diseases Testing Laboratory at Sidney Kimmel Medical College at Thomas Jefferson University.^30^ CSF protein was measured at local laboratories and retrieved from either patient medical records or ordered as part of the NDRD evaluation. Psychosine analyses were run at the laboratory of Dr. Michael Gelb at the University of Washington or at Mayo Clinic Laboratories – Rochester Main Campus, using punches from dried blood spots as per methods described by Gelb et al.^31^, ^32^, ^33^ Sample mixtures are passed through a high‐pressure liquid chromatography column prior to tandem mass spectrometry.^34^ The data are graphed longitudinally to illustrate changes over time.

### Other measures

MRI scans were obtained using the same protocol of sequences as described in Gupta et al., as part of the neuroradiologic evaluation.^35^ The MRIs were clinically evaluated by an experienced neuroradiologist, author of Zuccoli et al.^36^, ^37^, ^38^

Neurophysiologic testing included auditory brainstem responses (ABR) with a standard measurement of waves I‐V and was categorized as abnormal if any latency was prolonged more than 2 SD or any of the obligate waveforms (I, III, V) were absent. Nerve conduction velocity (NCV) motor responses were measured in the peroneal, tibial, and ulnar nerves, and sensory responses were measured in the sural and median nerves. Age specific norms were used. References for the normal ranges can be found in Parano et al. and Cruz et al.^39^, ^40^, ^41^ NCV studies were abnormal if they showed prolongation of distal and F‐wave latencies, low amplitude, or no evoked response. Skin temperature was obtained, and NCVs were obtained without sedation. Flash visual evoked potentials (VEPs) were abnormal if the P100 wave was absent.

### Statistical analysis

Basic descriptive statistics are reported for sex, race, ethnicity, follow‐up time, age at first symptoms, age of diagnosis and when applicable, age at the time of HSCT. Median age and ranges were calculated for each variable of interest to minimize the effect of outliers. To report on the prevalence of a sign or symptom of the disease, frequencies were calculated for age categories. The data were first examined using the following intervals: (0–3], (3–6], (6–9], (9–12], (12–18], (18–24], (24–36], and (36–60] months of age. Data were also summarized over larger periods, where the prevalence showed little variance across age periods.

The Kaplan–Meier method was used to estimate the median and overall survival time until May 1, 2022. Survival time was calculated as the age at which survivorship could be ascertained. Patients were censored at the last known survival date or the cutoff date for the analysis. Physical growth is depicted using the percentile calculations published by the CDC^42^ and is reported for each patient. Developmental growth is reported as age equivalent (AE) scores and graphed as age equivalence versus calendar age. Microcephalic and macrocephalic were defined as patients with 50% or more of their measures below the 3rd or above the 97th percentiles, respectively. To examine developmental milestone achievement, cumulative incidence graphs were calculated to illustrate the probability that a developmental skill had been acquired by a given age. Kaplan–Meier estimates were calculated in R 4.1.1 using the survminer package.

AE scores were used to determine improvements in all children including those with Quotients below the floor of the test.^44^ Using SAS 9.4, random effects models with random intercepts and slopes were used to estimate developmental trajectories for each group. For each model, the developmental AE score was the dependent variable and age (centered at 2.5 years), group, and the group x age interaction were the independent variables. Post hoc differences between the groups were calculated using the estimated coefficients from the models at 2.5 and 4.5 years of age. To better compare the gross motor development to typically developing children, we used a similar model to assess the PDMS Quotient score. The lowest possible Quotient score is <41, which causes truncation of the scores for the children with severe developmental delays, and thus, AE scores were used to assess disease progression. However, the asymptomatic HSCT group's scores are mostly higher than <41, and we also added Quotients for longitudinal tracking to capture more precisely the difference from the normal population.

Nonlinear (molecular) models were used to estimate the mean developmental trajectory of Gross Motor Function Measure – 88 (GMFM‐88) scores.

In order to track the progression of the disease, patients were divided into age groups based on the age at which their evaluations occurred. Patients who were evaluated once only contribute data to a single age group. Those followed longitudinally have multiple evaluations at different ages; data from such patients may be included at multiple time points.

## Results

### Patient characteristics and diagnosis

This study includes 137 children with infantile Krabbe disease. Patient characteristics are summarized in Tables 1 and S3.

**Table 1:** Summary of initial patient characteristics.

	Natural history *N* = 96		Symptomatic HSCT *N* = 18		Asymptomatic HSCT *N* = 23	
	Median	Range	Median	Range	Median	Range
Age at first evaluation (months)	8.4	0.8–47.8	6.7	4.2–23.0	0.8	0.1–40.1
Number of evaluations	2	1–9	3.5	1–10	7	1–11
Years followed	0	0–10.5	1.8	0–10.3	8.0	0–17.9
Age at first symptoms (months)	4	0–12	4.5	1.5–12	1	0.3–4.0
Age at diagnosis (months)	7	0–36	6.3	4–23	0	0–0.25
Age at HSCT (months)	–	–	8	5.1–23.7	1.1	0.6–4.9
Initial GALC levels	0.05	0–1.10	0.03	0–0.12	0.07	0–0.23
Initial CSF protein levels	199	34–594	219	83–571	191	39–528
Initial psychosine levels	19.7	0.2–82.3	38.9	5.0–54.0	6.3	0.5–29.1

One hundred and thirty‐seven children with infantile‐onset Krabbe disease were included in this study (68 males and 69 females). Of the 137 patients, 96 did not receive HSCT and were considered natural history patients and 41 underwent HSCT, of whom 18 were symptomatic and 23 were asymptomatic at the time of transplantation. Eighty‐seven patients were followed longitudinally with the median number of evaluations as 2 for the natural history patients, 3.5 for the symptomatic HSCT patients, and 7 for the asymptomatic HSCT patients.

One hundred and thirteen patients were diagnosed after symptoms. Nineteen patients were identified and diagnosed because of family history, and five were diagnosed via NBS. One baby died during the transplant at an outside institution and only had a baseline assessment prior to HSCT and is included in the natural history (NH) group. A total of 448 evaluations were conducted, including 211 NH, 86 Symptomatic (Sympt) HSCT, and 151 Asymptomatic (Asympt) HSCT (Tables 1 and S4).

### Neonatal history and initial symptoms

Several neonatal complications were reported. None of these variables were above those reported in the normal population of newborns (Table S5).

Initial symptoms were defined as a change, causing parental and/or physician concern. Data on initial symptoms were available in 117 patients. Irritability was the most common (*n* = 66; 56%) followed by developmental delay or loss of acquired milestones (*n* = 58; 46%), feeding or swallowing difficulties (*n* = 55; 46%), spasticity (*n* = 51; 43%), gastroesophageal reflux (*n* = 40; 34%), abnormal movements or seizures (*n* = 22; 18%), failure to thrive (*n* = 18; 15%), and hypotonia (*n* = 6; 5%). Assessment demonstrated that the median age of onset for irritability was 4 months (range: 0–14 months), with 80 (71%) patients presenting with irritability by 8 months of age.

### Survival

**Figure 1:**
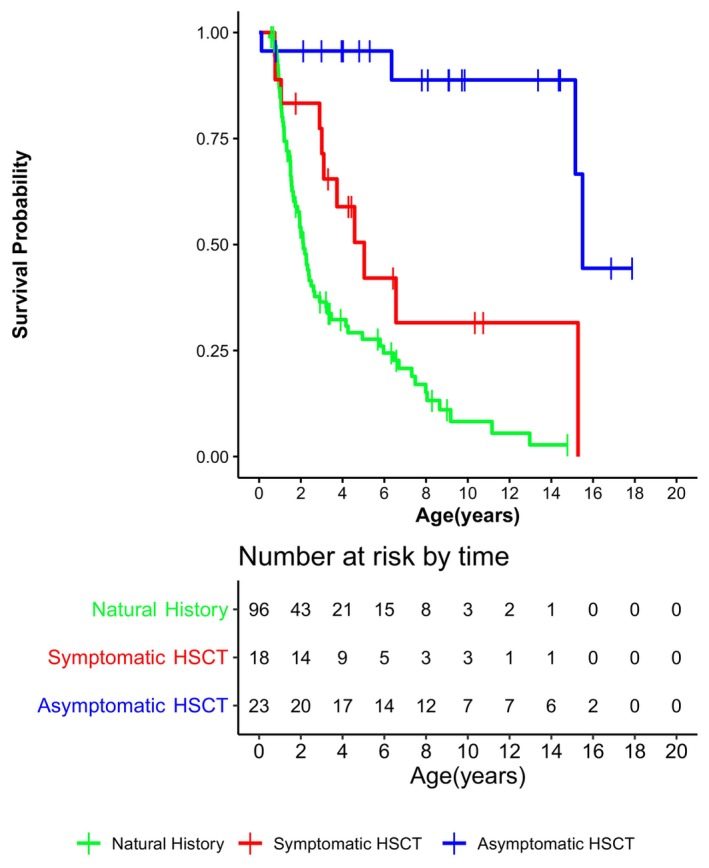
Kaplan–Meier survival curves of natural history patients in green, symptomatic HSCT patients in red, and asymptomatic HSCT patients in blue. Hematopoietic stem cell transplantation is abbreviated to “HSCT”.

As of May 1, 2022, 50 of the 137 patients are surviving. The median survival probability for the NH group was 2.2 years, 5.0 for the Sympt HSCT group, and 15.5 for the Asympt HSCT group (Fig. 1). The Asympt HSCT group shows significantly higher survival probabilities than the NH and Sympt HSCT groups (*p* < 0.001).

### Growth

**Figure 2:**
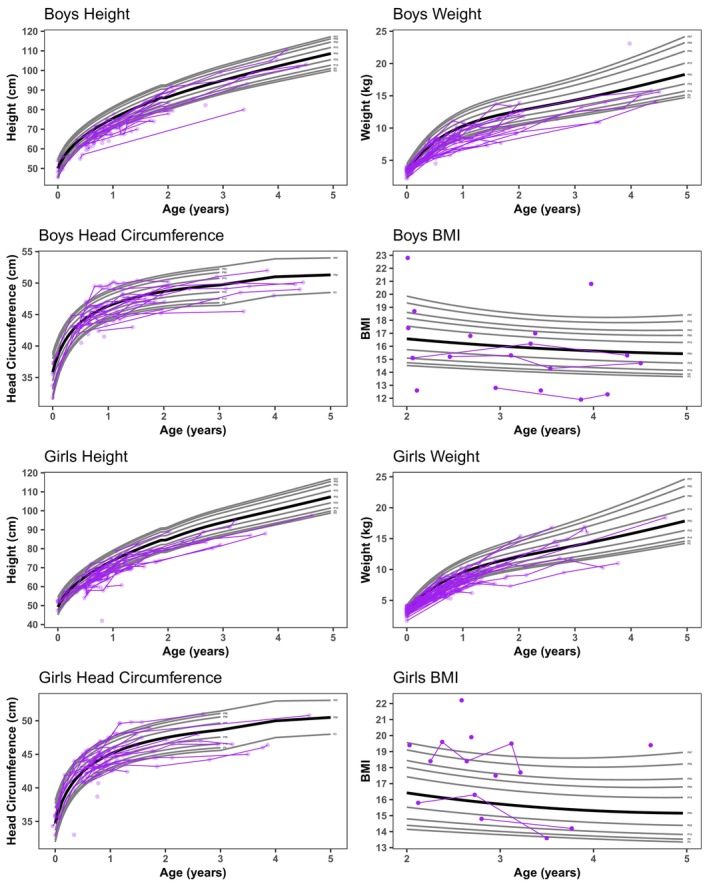
Height, weight, head circumference, and body mass index (BMI) of natural history patients in purple.

**Figure 3:**
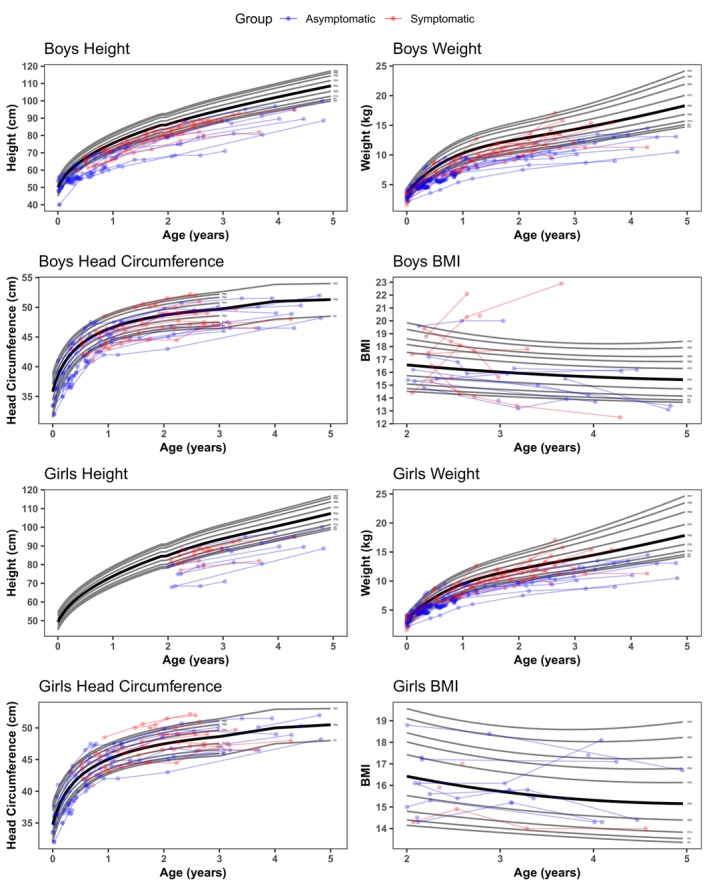
Height, weight, head circumference, and body mass index (BMI) of asymptomatic HSCT patients in blue and symptomatic HSCT patients in red. Hematopoietic stem cell transplantation is abbreviated to “HSCT”.

The NH patients were below the normal range in height and weight, but only four patients reported body mass index (BMI) values below the fifth percentile (Table 2, Fig. 2). For transplanted patients, most were below the fifth percentile for height and weight, though BMI was closer to normal (Fig. 3). Microcephaly rates were similar across the groups, ranging from 11% to 17% of the patients. Macrocephaly was observed in only 2% of the NH patients and in none of the HSCT patients.

**Table 2:** Summary of the growth percentiles in natural history, symptomatic HSCT, and asymptomatic HSCT patients, indicating percentages below the third percentile for head circumference and below the fifth percentile for height, weight, and body mass index.

	Head circumference (<3rd percentile)	Height (<5th percentile)	Weight (<5th percentile)	BMI (<5th percentile)
Natural history	12%	56%	59%	4%
Symptomatic HSCT	11%	67%	56%	22%
Asymptomatic HSCT	17%	91%	96%	22%

Microcephaly was defined as head circumference below the third percentile and macrocephaly as head circumference above the 97th percentile, in accordance with the CDC.

### Feeding and digestive issues

Gastrointestinal problems, like constipation and gastroesophageal reflux (GER), were present in most patients. Constipation occurred in 29% of NH patients, increasing to 90% by 36 months. Feeding difficulties were documented by 2 months of age and continued to be abnormal. Asympt HSCT patients also had feeding problems but were less prevalent. GER was present in 80%–100% of NH patients from birth‐8 months and declined at 9 months likely due to treatment with Nissen fundoplication and anti‐reflux medications though eventually, reflux increased by 36–60 months. Sympt HSCT patients had less GER from birth‐6 months but increased to 25%–60% through 60 months. Asympt HSCT patients had GER, ranging from 0 to 11% across the age groups.

For the NH and Sympt HSCT groups, swallowing difficulties were reported from 3 months of age at 76% and affected 100% of both groups by 18–23 months. Conversely, the Asympt HSCT patients had lower rates of swallowing difficulties across the age range. All the NH and Sympt HSCT patients required a feeding tube (FT) by a median age of 10.8 and 14.8 months, respectively. Data were available for 21 of the 23 Asympt HSCT patients and of these, 9 never required a FT.

As early as 3 months of age, NH patients needed to be suctioned to manage their secretions, and this requirement increased with age. The Sympt HSCT group had a similar need to NH by 36 months, while the Asympt HSCT group did not need suctioning from birth‐60 months of age (Table S6).

## Developmental Outcomes

### Overall gross motor functioning

**Figure 4:**
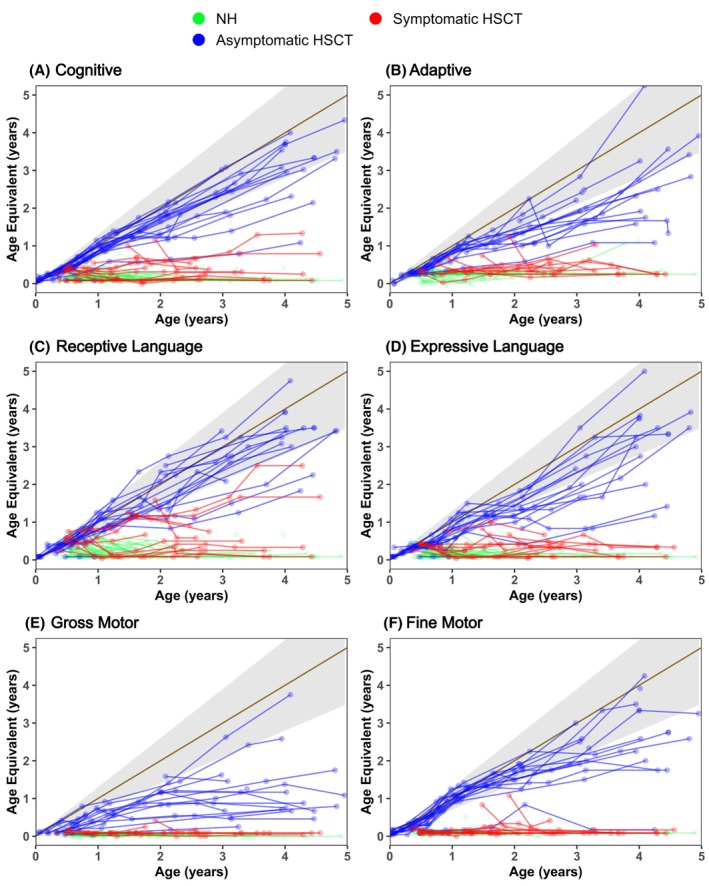
Neurodevelopmental outcomes across six domains for natural history patients in green, symptomatic HSCT patients in red, and asymptomatic HSCT patients in blue. Gray shading indicates normal development. The *x*‐axis indicates the actual age of the patient, and the *y*‐axis indicates the developmental equivalent age. Age equivalent score can be calculated indicating the developmental age at which a patient is functioning. For example, a 36‐month patient with a score of 22 would have the skills of an average 22‐month‐old. Hematopoietic stem cell transplantation is abbreviated to “HSCT”.

General gross motor (GM) development was measured using the Peabody Developmental Motor Scales, PDMS‐II (see Fig. 4).^28^

**Figure 5:**
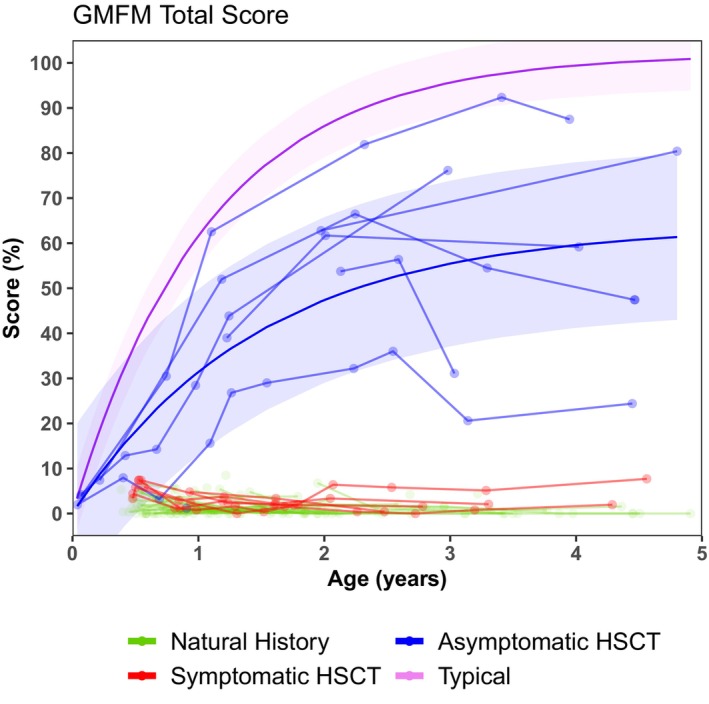
Longitudinal total gross motor function measure (GMFM) scores of natural history patients in green, symptomatic HSCT patients in red, and asymptomatic HSCT patients in blue. Typical patients are indicated in purple. Hematopoietic stem cell transplantation is abbreviated to “HSCT.”

The NH and Sympt HSCT patients showed no difference in their GMFM‐88 scores in that neither scored above 10%. However, the Asympt HSCT patients showed varying degrees of improved function, though no one reached 100%, which would be typical at 5 years of age (Fig. 5). Only one patient reached 90%, and the rest scored between 25% and 80%. GM development after 5 years of age in infantile KD patients that were transplanted in the first 7 weeks of life is reported in Wright et al.^43^

### Sitting

**Figure 6:**
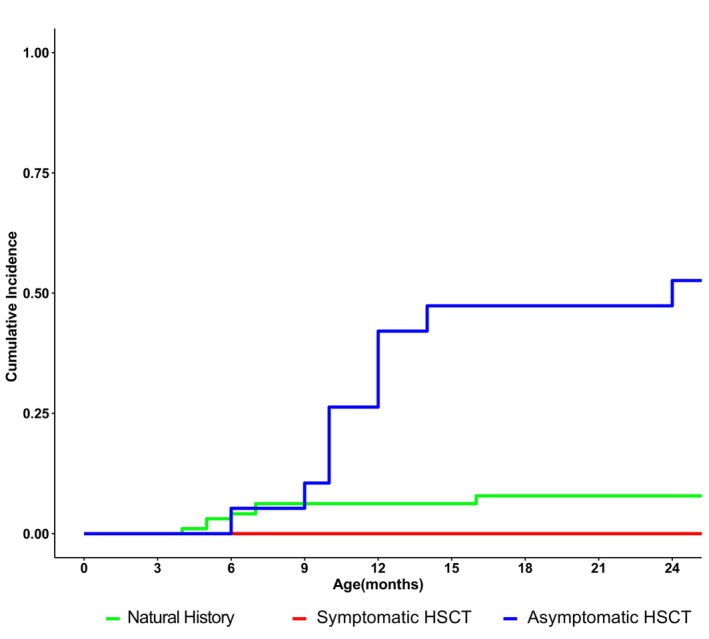
Cumulative incidence of sitting without assistance through 24 months of age for natural history patients in green, symptomatic HSCT patients in red, and asymptomatic HSCT patients in blue. Hematopoietic stem cell transplantation is abbreviated to “HSCT.”

Relying on both parent reports and clinical observation, only 10% of the NH group achieved sitting independently (Fig. 6). None of the Sympt HSCT patients ever sat independently. As for the Asympt HSCT group, 42% achieved it by 12 months, and this percentage increased to 52% by 24 months (Table S6).

### Walking

**Figure 7:**
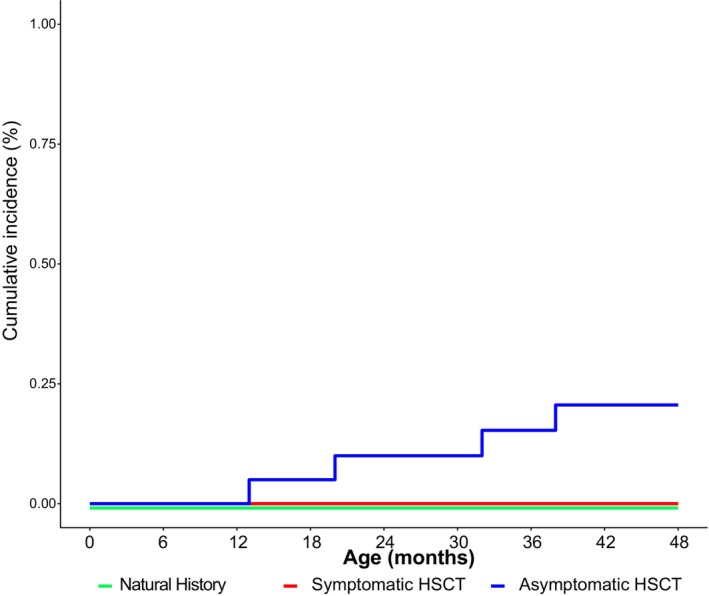
Cumulative incidence of walking without assistance through 48 months of age for natural history patients in green, symptomatic HSCT patients in red, and asymptomatic HSCT patients in blue. Hematopoietic stem cell transplantation is abbreviated to “HSCT.”

Mobility became significantly more limited as the disease progressed with 91% of the NH patients developing quadriparesis between 12 and 14 months of age, and 100% by 24 months. Axial hypotonia was observed in 46% of the NH patients aged 3–5 months, affecting 100% of patients by 18–60 months. Among the Sympt HSCT patients, axial hypotonia increased from 50% to 100% by 18 months. While not reaching 100%, the Asympt HSCT patients showed similar trunk weakness, increasing from 50% at 3–6 months to 71% by 18 months and ranging from 75% to 89% thereafter (Table S6). No one walked without assistance in the NH and Sympt HSCT groups, though 4 of 23 did in the Asympt HSCT group (Fig. 7, Table S7).

### Neuromuscular and orthopedic findings

In the NH group, appendicular spasticity of the upper and/or lower extremities was present in 97% of patients evaluated between birth and 12 months, decreasing to 74% after 12 months. Similarly, nearly all Sympt HSCT patients were affected. Spasticity in the Asympt HSCT group was lower but increased over time, especially during periods of rapid growth. Both the NH and Sympt HSCT groups showed higher rates of fisting at younger ages that decreased over time. The Asympt HSCT group showed lower rates across all ages.

Abnormal deep tendon reflexes (DTR) were present in almost all NH evaluations, with 83%–100% across the age periods. Similar results were observed in the Sympt HSCT group with 80%–100%. However, the Asympt HSCT group reported only 27% from 0 to 3 months, and this percentage increased to 82% after 12 months. Bulging fontanelle was reported in 23% of the NH evaluations from 0 to 11 months and 42% from 12 to 60 months; one case required a ventriculoperitoneal shunt.

Due to spasticity, orthopedic complications developed in NH patients. Hip asymmetry during physical examination prompted x‐rays to confirm hip dislocation or hip subluxation, and it affected 100% of the cohort after 36 months and ranged from 17% to 50% in the Sympt HSCT group. The Asympt HSCT group was rarely affected until 36–60 months with 18% of evaluations reporting hip asymmetry. Scoliosis was observed in 24% of NH patients between 9 and 11 months, increasing to 100% after 36 months. Among the Sympt HSCT patients, scoliosis was reported in 60% of evaluations after 18 months; in contrast, only 17% of Asympt HSCT evaluations had scoliosis (Table S6).

### Staring episodes and seizures

Staring episodes began at 3–6 months in the NH group with 27% reporting their occurrence. This number increased to 85% by 24 months of age. Both the Sympt and Asympt HSCT groups reported fewer staring episodes than the NH one. Clinical seizures were observed as early as 3–5 months for the NH patients and increased from 7% to 28% at 12–60 months. The Sympt HSCT patients reported clinical seizures, ranging from 0% to 25% and only 4 out of 20 Asympt HSCT patients reported seizures (20%), which started at 6, 20, 21, and 23 months of age (Table S6).

### Vision and hearing

NH patients had visual tracking difficulties early in the disease process with 75% remaining affected at all age points (Table S6). Sympt HSCT patients ranging from 20% to 50% of evaluations had difficulties. Asympt HSCT patients showed abnormalities before 9 months, and no new tracking issues were reported thereafter. Abnormal eye movements, like jerky or fluttering oculomotor movements, increased from 10% for the 3‐6‐month period to 75% by 24 months. Only two Sympt HSCT patients reported abnormal eye movements at 12 months, and no Asympt HSCT patients presented with them. Across groups, 63% had abnormal VEPs between 0 and 3 months, including a 5‐day‐old baby. Between 3 and 24 months, 50% of the Sympt HSCT group, 32% of the NH group, and 15% of the Asympt HSCT group had abnormal VEPs.

Although 2% of parents reported hearing loss, auditory neuropathy, identified through ABR, revealed that 73% of patients evaluated between 0 and 3 months had abnormalities in Waves I, III, or V, and this percentage increased to 90% by 60 months. Among the Sympt HSCT patient evaluations, 95% were abnormal during 3–24 months and 67% during 24–60 months. Asympt HSCT patients remained abnormal with 71%–82% of evaluations reporting abnormalities (Table S6). The abnormalities were mostly due to auditory neuropathy (prolonged conduction but normal speech recognition at 20 db).

### Neurodevelopmental function

NH patients showed little to no development across all six developmental domains (Figs. 4 and 5, S1, Table S8). The Sympt HSCT group scored similarly on 5 of the domains but scored slightly better on receptive language (*p* = 0.006). A few Sympt HSCT children showed gains in receptive language, though never advanced beyond an age equivalent of 3 years.

Conversely, the Asympt HSCT group showed continuous skill acquisition that in some cases fell within the range of normal development (Fig. 4). Most of the children showed normal or near‐normal acquisition of skills in cognitive development and receptive language. However, the group showed a leveling off of gross motor skills below a 2‐year‐age equivalent, with only 2 out of 10 children performing in the normal range at 4 years of age (Figs. S2 and S3, Table S8). The group performed slightly better on fine motor skills, though most slowed or plateaued around 18–24 months (Fig. 4). However, at 4 years of age, 3 out of 11 were developing within the normal range. The Asympt HSCT group showed higher scores and greater gains over time than both the NH and Sympt HSCT groups for all domains (all *p* < 0.001) (Table S9).

## Neuroradiologic and Neurophysiologic Testing

### Brain magnetic resonance imaging

Abnormalities on MRI were detected on 71% (15/21) of the NH/pretransplant scans during 0–3 months. The most common abnormalities were increased T2 signaling in the periventricular white matter and involvement of the corticospinal tracts and corpus callosum. Ninety‐seven percent of the NH patients' scans were abnormal at 3–24 months (*n* = 62) and 100% abnormal after 24 months (*n* = 13). The Sympt HSCT group only had scans after 3 months of age, and 94% of the patients were abnormal from 3 to 24 months (*n* = 17) and 100% abnormal thereafter (*n* = 12). In the Asympt HSCT group, 74% of scans were abnormal from 3 to 24 months (*n* = 19), and 92% after 24 months (*n* = 12).

### Nerve conduction velocity

Of the 91 patients with initial NH NCVs, only 5 were normal. Of the 15 Sympt HSCT patients with post‐HSCT data, one was abnormal pre‐HSCT but became normal by 31 months of age. Of the 17 Asympt HSCT patients with post‐HSCT data, 3 were abnormal pre‐HSCT and became normal post‐HSCT. However, 2 of the 3 slowly became abnormal by 27 and 20 months, while the third remained normal at their last evaluation at 41 months.

### Galactocerebrosidase measurement

The initial GALC median value for the untreated patients was 0.05 (range: 0.0–1.10) (Table S10a). The one patient with a high GALC of 1.10 was suspected of having Saposin A deficiency because they developed rapid disease progression and died. The Sympt HSCT and Asympt HSCT groups had nearly identical median post‐HSCT values with a median of 2.6 (range: 0.10–5.90). Detailed GALC levels by time since HSCT are reported in Table S10b.

## Biomarkers

### Psychosine

**Figure 8:**
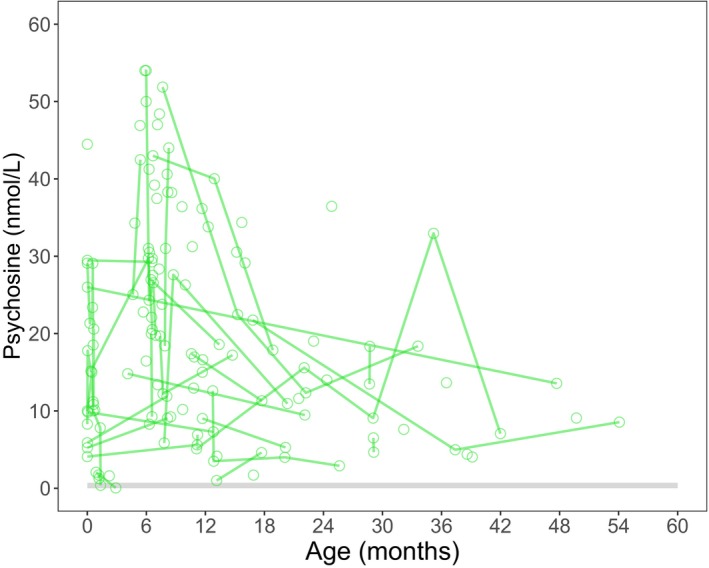
Longitudinal psychosine values for natural history patients.

**Figure 9:**
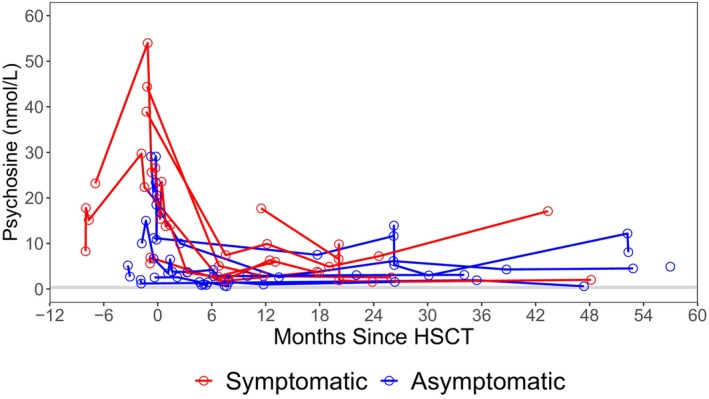
Longitudinal post‐transplant psychosine values of symptomatic HSCT (red) and asymptomatic HSCT (blue) patients. Hematopoietic stem cell transplantation is abbreviated to “HSCT.”

Median pretreatment values of psychosine were 22.3 (range: 1.0–82.3) for the NH group (Fig. 8), 28.1 (range: 4.1–54.0) for the Sympt HSCT group, and 6.3 (range: 1.0–29.1) for the Asympt HSCT group. At 21–27 months post‐HSCT, the median for the Sympt HSCT group was 2.5 (range: 1.1–7.5) and 4.1 (range: 1.4–9.6) for the Asympt HSCT group (Fig. 9). Detailed psychosine levels by age for NH and HSCT patients are reported in Tables S10a and S10b, respectively.

### CSF protein

Initial CSF Protein median value for the NH patients was 199 (range: 34–594), 219 (range: 83–571) for the Sympt HSCT patients, and 191 (39–528) for the Asympt HSCT patients. At 21–27 months post‐HSCT, the median CSF protein levels for the Sympt HSCT group were 204 (range: 143–212) and 143 (range: 86–198) for the Asympt HSCT one. Detailed GALC levels by age for NH and HSCT patients are reported in Tables S10a and S10b, respectively.

## Discussion

In this study, we prospectively evaluated a large cohort of patients with KD onset between 0 and 12 months. All patients were evaluated at the same center using standardized protocols. This improves reliability and variability of results, making it the largest of its kind. We introduced clinical endpoints based on 137 patients evaluated over 20 years, including untreated and transplanted ones. These endpoints can be used to compare results of future therapies. According to our findings, the Asympt HSCT patients had better functional outcomes overall as compared to the Sympt HSCT and NH patients. Our results indicate that Asympt HSCT patients had the best functional outcomes, including cognitive, motor, and adaptive development as well as better vision and hearing and less feeding difficulties.

It was not until 2004 that it was noted that patients who were symptomatic had similar outcomes as untreated patients except survival was longer.^9^ From this point on, only minimally symptomatic patients were transplanted.

In Bascou et al., we documented the natural course of progression in 35 patients with onset between 6 and 36 months, and in Beltran‐Quintero et al., we characterized disease manifestation in 88 patients with onset between 0 and 5 months.^1^, ^18^ Given our re‐classification of patients with onset ≤12 months as infantile, we combined data from both studies as well as increased our cohort with additional patients to better understand the clinical manifestations of the infantile‐onset as a group. This is the largest single‐site prospective study that characterizes the infantile‐onset as a group which constitutes 85%–90% of Krabbe cases, including geographically diverse patients from at least 41 states and 14 countries and a range of racial backgrounds.

In Bascou et al., we found that patients with onset ≤12 months had a more aggressive phenotype similar to patients with early onset.^1^ NBS patients that were at high risk were closely monitored with detailed physical and neurological examinations, brain MRIs, VEPs, ABRs, and developmental testing that enabled us to comprehensively describe their phenotype. We also re‐demonstrated that most asymptomatic or minimally symptomatic patients from birth to 3 months of age had abnormal MRIs, ABRs, and NCVs, highlighting the need for early evaluations and monitoring during the first year of life.

Nutrition and growth are areas affecting this population, and therefore, we looked at feeding and growth in detail. Twenty‐two percent of HSCT patients were below the fifth percentile in BMI while only 4% of the NH patients were below the fifth percentile (Table 2). Although chemotherapy does not stunt growth in other therapies, busulfan‐based preconditioning regimens for HSCT have been shown to slow growth rates in very young children.^44^, ^45^ Interestingly, the BMIs showed the expected variability in all groups. The NH patients' growth was mostly below the 50th percentile; however, they followed normal variability in BMI as a group.

Of the 12 Asympt HSCT patients that required feeding tubes, some relied on them for supplemental nutrition, while others had trouble swallowing foods and/or liquids. Our practice was to recommend feeding tubes early to prevent complications; however, variability in the timing of G‐tube placement existed due to some surgeons requiring the barium swallow study to show evidence of aspiration and delayed scheduling. Some patients opted for a nasogastric tube. Although the Asympt HSCT group did not need suctioning up to 60 months of age, some of these patients were put on medications that partially dried their secretions. This accounts for the 5% of Asympt HSCT patients that required suctioning between 36 and 60 months of age.

Regarding NBS, we believe it is important to identify high‐risk patients and refer them immediately to treatment centers, where they can be further evaluated by expert teams. In this study, we show that the treatment timing is imperative for best functional outcomes. A few weeks can make a difference between being eligible or ineligible for transplant. Once motor skills are lost, they are not regained. Several challenges are encountered by physicians evaluating these patients given the rarity of the disease. Multidisciplinary resources with the needed expertise are locally scarce, so families often have to travel to centers of excellence that are out‐of‐state. Approving out‐of‐network insurance, scheduling multiple assessments on short notice, and coordinating parent travel require a dedicated team within the hospital and additional financial support.

Leukodystrophy centers across the United States, mostly funded by family foundations and NIH‐funded rare disease networks, are working together to standardize care and help families shorten the time to treatment. Evaluating patients comprehensively to confirm diagnosis and phenotype as early as possible is critical for providing appropriate counseling to parents about potential outcomes of transplant. The NH group (too symptomatic to benefit from HSCT) was identified just 3–4 weeks after the minimally symptomatic group and 6 to 6.5 months later than the asymptomatic patients that benefitted from transplant This underscores the urgency to refer patients before they become ineligible for treatment. Additionally, timely transplant maximizes developmental outcomes by preventing irreversible damage.

It is important to emphasize that the decision to transplant is not based on a single parameter but on a combination of findings including biochemical, neuroradiological, neurophysiological, and neurodevelopmental evaluations. We discuss what tests are most important in Escolar et al., or the initial evaluation and follow‐up of KD patients, and emphasize the importance of comprehensively observing and accurately interpreting all tests, including physical examination signs and laboratory values.^10^ One of the biggest challenges to early diagnosis is that initial manifestations of KD can be overlooked due to lack of familiarity among providers and parents and mistaken for symptoms that are very prevalent in the population, such as feeding difficulties or irritability at 4 months that present similar to colic. Additionally, general providers may not have adequate training in the neurological examination of a newborn, including atypical muscle tone, and evaluating abnormal feeding, contributing to under‐ or misdiagnosis. Lastly, some providers struggle determining what clinical evaluations are needed to diagnose and transplant a patient. It is our recommendation that patients identified through NBS are immediately referred to a transplant center that has significant multidisciplinary experience in this disease.

Another point of confusion is distinguishing between asymptomatic and symptomatic infantile KD patients. Asymptomatic patients can have a normal physical examination and behavior but show abnormalities on MRI and neurophysiologic tests. Therefore, in order to be considered “symptomatic,” infantile KD patients need to be abnormal in more than one criterion in addition to having low GALC levels and/or increased psychosine, as explained in the Supplementary Text.

Based on this study, we consider the motor endpoints to be the most meaningful clinical parameters for use as endpoints in clinical trials since currently, even those patients that receive HSCT asymptomatically still develop motor disease due to progressive peripheral neuropathy.

Our study has limitations, including difficulty obtaining outside medical records and reaching out to patients for longitudinal follow‐up once they became too symptomatic to travel to our site. In addition, some of our patients rely on adaptive equipment, and as a result, it was challenging to capture their true developmental level of independence. Furthermore, some children with HSCT following their baseline assessment were not available for longitudinal follow‐up. While we had a recommended schedule, it was left to the ability of the patients to comply with visits, and there were many factors influencing this, including financial, health of the patient, ability for the parents to request off work, and insurance approval. Considering asymptomatic and minimally symptomatic patients with less severe, slower progressing phenotypes are often the best candidates for HSCT, this may have caused an implicit bias regarding the patients who were followed longitudinally. Because certain symptoms were treated following baseline visits, such as reflux, feeding and swallowing difficulties, diarrhea, and constipation, the incidence of these symptoms in our population may be slightly decreased with respect to the disease's natural history.

Despite these limitations, this study remains the most standardized, longest, and largest of its kind. Given its longitudinal and prospective follow‐up with standardized protocols for physical examinations and neurodevelopmental assessments, we were able to reconcile our past characterization of the infantile‐onset, further validating the re‐classification. Based on our results, we confirm the rapid progression of the infantile‐onset presenting from birth to 12 months, underscoring the need to re‐classify infantile KD to better distinguish patients with severe phenotype and provide early management options. Meaningful outcomes are those that improve the quality of life of a patient, and therefore, we chose to use the cumulative incidences of sitting and walking independently. Additionally, we describe the longitudinal progression with the GMFM‐88 and the PDMS‐II, both robust and standardized measures of motor development. These tests clearly captured both the neurodegenerative process and the transplant treatment effects in the motor area.

## Author Contributions

MLE conceptualized, planned the study, provided oversight, and obtained funding to support family travel and staff from 2000 until 2022. She examined patients, provided mentorship to first co‐authors and second and third authors, and edited the manuscript. MDP designed the methodology and statistical analysis, and provided oversight of the data for the last 19 years and contributed to editing of the manuscript. She provided mentorship to first co‐authors and second and third authors. MAL extracted data and drafted the initial manuscript. MRG performed data extraction and finalized writing. MLB facilitated data collection and analysis from the 0‐ to 6‐month cohort. ETB performed data extraction and contributed to writing.

## Conflict of Interest Statement

MLE is the Chief Medical Officer at Forge Biologics with an ownership interest greater than 5% and maintains full time employment since February of 2022. MDP maintains full time employment with Forge Biologics since October of 2022. The other authors have no conflicts to disclose.

## Supporting information


Data S1.

Figure S1.

Figure S2.

Figure S3.

Table S1.

Table S3.

Table S4.

Table S5.

Table S6.

Table S7.

Table S8.

Table S9.

Table S10a.

Table S10b.

## Data Availability

The data that support the findings of this study are available from the corresponding author upon reasonable request.

## References

[1] Bascou N , DeRenzo A , Poe MD , Escolar ML . A prospective natural history study of Krabbe disease in a patient cohort with onset between 6 months and 3 years of life. Orphanet J Rare Dis. 2018;13(1):126. 30089515 10.1186/s13023-018-0872-9 PMC6083585

[2] Suzuki K . Globoid cell leukodystrophy (Krabbe's disease): update. J Child Neurol. 2003;18(9):595‐603. 14572137 10.1177/08830738030180090201

[3] Castelvetri LC , Givogri MI , Zhu H , et al. Axonopathy is a compounding factor in the pathogenesis of Krabbe disease. Acta Neuropathol. 2011;122(1):35‐48. 21373782 10.1007/s00401-011-0814-2 PMC3690521

[4] Moser HW . Peripheral nerve involvement in Krabbe disease: a guide to therapy selection and evaluation. Neurology. 2006;67(2):201‐202. 16864808 10.1212/01.wnl.0000231531.73713.a9

[5] Escolar ML , West T , Dallavecchia A , Poe MD , LaPoint K . Clinical management of Krabbe disease. J Neurosci Res. 2016;94(11):1118‐1125. 27638597 10.1002/jnr.23891

[6] Wenger DA , Rafi MA , Luzi P , Datto J , Costantino‐Ceccarini E . Krabbe disease: genetic aspects and progress toward therapy. Mol Genet Metab. 2000;70(1):1‐9. 10833326 10.1006/mgme.2000.2990

[7] Sakai N . Pathogenesis of leukodystrophy for Krabbe disease: molecular mechanism and clinical treatment. Brain and Development. 2009;31(7):485‐487. 19332366 10.1016/j.braindev.2009.03.001

[8] Lim ZY , Ho AY , Abrahams S , et al. Sustained neurological improvement following reduced‐intensity conditioning allogenic hematopoietic stem cell transplantation for late‐onset Krabbe disease. Bone Marrow Transplant. 2008;41(9):831‐832. 18246117 10.1038/sj.bmt.1705984

[9] Escolar ML , Poe MD , Provenzale JM , et al. Transplantation of umbilical‐cord blood in babies with infantile Krabbe's disease. N Engl J Med. 2005;352(20):2069‐2081. 15901860 10.1056/NEJMoa042604

[10] Escolar ML , Poe MD , Martin HR , Kurtzberg J . A staging system for infantile Krabbe disease to predict outcome after unrelated umbilical cord blood transplantation. Pediatrics. 2006;118(3):e879‐e889. 16923928 10.1542/peds.2006-0747

[11] Wasserstein MP , Andriola M , Arnold G , et al. Clinical outcomes of children with abnormal newborn screening results for Krabbe disease in New York State. Genet Med. 2016;18(12):1235‐1243. 27171547 10.1038/gim.2016.35

[12] Orsini JJ , Saavedra‐Matiz CA , Gelb MH , Caggana M . Newborn screening for Krabbe's disease. J Neurosci Res. 2016;94(11):1063‐1075. 27638592 10.1002/jnr.23781 PMC5328187

[13] Lantos JD . Dangerous and expensive screening and treatment for rare childhood diseases: the case of Krabbe disease. Dev Disabil Res Rev. 2011;17(1):15‐18. 22447750 10.1002/ddrr.133 PMC4014301

[14] Kwon JM , Matern D , Kurtzberg J , et al. Consensus guidelines for newborn screening, diagnosis and treatment of infantile Krabbe disease. Orphanet J Rare Dis. 2018;13(1):30. 29391017 10.1186/s13023-018-0766-x PMC5796396

[15] Escolar ML , Kiely BT , Shawgo E , et al. Psychosine, a marker of Krabbe phenotype and treatment effect. Mol Genet Metab. 2017;121(3):271‐278. 28579020 10.1016/j.ymgme.2017.05.015 PMC5548593

[16] Wenger DA , Suzuki K , Suzuki Y , Suzuki K . Galactosylceramide lipidosis: globoid cell leukodystrophy (Krabbe disease). In: Scriver CR , Sly WS , Childs B , et al., eds. The Metabolic and Molecular Bases of Inherited Disease. 8th ed. McGraw‐Hill; 2001:3669‐3694.

[17] Hagberg B , Sourander P , Svennerholm L . Diagnosis of Krabbe's infantile leucodystrophy. J Neurol Neurosurg Psychiatry. 1963;26(3):195‐198. 13951860 10.1136/jnnp.26.3.195 PMC1074213

[18] Beltran‐Quintero ML , Bascou NA , Poe MD , et al. Early progression of Krabbe disease in patients with symptom onset between 0 and 5 months. Orphanet J Rare Dis. 2019;14(1):46. 30777126 10.1186/s13023-019-1018-4 PMC6378723

[19] Shao YH , Choquet K , La Piana R , et al. Mutations in GALC cause late‐onset Krabbe disease with predominant cerebellar ataxia. Neurogenetics. 2016;17(2):137‐141. 26915362 10.1007/s10048-016-0476-2

[20] Morse LE , Rosman NP . Myoclonic seizures in Krabbe disease: a unique presentation in late‐onset type. Pediatr Neurol. 2006;35(2):154‐157. 16876017 10.1016/j.pediatrneurol.2006.02.004

[21] Pavuluri P , Vadakedath S , Gundu R , Uppulety S , Kandi V . Krabbe disease: report of a rare lipid storage and neurodegenerative disorder. Cureus. 2017;9(1):e949. 28168127 10.7759/cureus.949 PMC5289898

[22] Wenger DA , Escolar ML , Luzi P , Rafi MA . Krabbe disease (globoid cell leukodystrophy). Scriver's the Online Metabolic and Molecular Bases of Inherited Disease (OMMBID). McGraw Hill; 2013. doi:10.1036/ommbid.178

[23] Duffner PK , Jalal K , Carter RL . The Hunter's Hope Krabbe family database. Pediatr Neurol. 2009;40(1):13‐18. 19068248 10.1016/j.pediatrneurol.2008.08.011

[24] Yoon IC , Bascou NA , Poe MD , Szabolcs P , Escolar ML . Long‐term neurodevelopmental outcomes of hematopoietic stem cell transplantation for late‐infantile Krabbe disease. Blood. 2021;137(13):1719‐1730. 33150395 10.1182/blood.2020005477 PMC8020262

[25] Martin HR , Poe MD , Reinhartsen D , et al. Methods for assessing neurodevelopment in lysosomal storage diseases and related disorders: a multidisciplinary perspective. Acta Paediatr. 2008;97(457):69‐75. 18339192 10.1111/j.1651-2227.2008.00651.x

[26] Mullen EM . Mullen Scales of Early Learning. AGS ed. American Guidance Service. Inc.; 1995.

[27] Russell DJ , Rosenbaum PL , Wright M , Avery LM . Gross Motor Function Measure (GMFM and GMFM‐88) User's Manual. 2nd ed. MacKeith Press; 2013.

[28] Folio MR , Fewell RR . Peabody developmental motor scales. 2nd ed. Pro‐Ed; 2000.

[29] Burger‐Caplan R , Saulnier CA , Sparrow SS . Vineland Adaptive Behavior Scales. Encyclopedia of clinical neuropsychology. Springer International Publishing; 2018:1‐5.

[30] Chen YQ , Wenger DA . Galactocerebrosidase from human urine: purification and partial characterization. Biochim Biophys Acta. 1993;1170(1):53‐61. 8399327 10.1016/0005-2760(93)90175-9

[31] Gelb MH , Turecek F , Scott CR , Chamoles NA . Direct multiplex assay of enzymes in dried blood spots by tandem mass spectrometry for the newborn screening of lysosomal storage disorders. J Inherit Metab Dis. 2006;29(2‐3):397‐404. 16763908 10.1007/s10545-006-0265-4 PMC2488386

[32] Gelb MH . Newborn screening for lysosomal storage diseases: methodologies, screen positive rates, normalization of datasets, second‐tier tests, and post‐analysis tools. Int J Neonatal Screen. 2018;4(3):23. 30882045 10.3390/ijns4030023 PMC6419971

[33] Gelb MH , Lukacs Z , Ranieri E , Schielen P . Newborn screening for lysosomal storage disorders: methodologies for measurement of enzymatic activities in dried blood spots. Int J Neonatal Screen. 2019;5(1):1. 30957052 10.3390/ijns5010001 PMC6448570

[34] Gelb MH , Basheeruddin K , Burlina A , et al. Liquid chromatography‐tandem mass spectrometry in newborn screening laboratories. Int J Neonatal Screen. 2022;8(4):62. 36547379 10.3390/ijns8040062 PMC9781967

[35] Gupta A , Poe MD , Styner MA , Panigrahy A , Escolar ML . Regional differences in fiber tractography predict neurodevelopmental outcomes in neonates with infantile Krabbe disease. Neuroimage Clin. 2014;7:792‐798. 25844309 10.1016/j.nicl.2014.09.014 PMC4375637

[36] Zuccoli G , Narayanan S , Panigrahy A , Poe MD , Escolar ML . Midbrain morphology reflects extent of brain damage in Krabbe disease. Neuroradiology. 2015;57(7):739‐745. 25859833 10.1007/s00234-015-1523-7

[37] Hwang M , Zuccoli G , Panigrahy A , Rodriguez D , Poe MD , Escolar ML . Thickening of the cauda equina roots: a common finding in Krabbe disease. Eur Radiol. 2016;26(10):3377‐3382. 27137647 10.1007/s00330-016-4233-6

[38] Zuccoli G , Kim A , Poe M , Escolar ML . Spontaneous third ventriculostomy in Krabbe disease. Pediatr Neurol. 2020;108:99‐105. 32197817 10.1016/j.pediatrneurol.2019.11.014 PMC7263959

[39] Parano E , Uncini A , De Vivo DC , Lovelace RE . Electrophysiologic correlates of peripheral nervous system maturation in infancy and childhood. J Child Neurol. 1993;8(4):336‐338. 8228028 10.1177/088307389300800408

[40] Martinez AC , Ferrer MT , Conde MC , Bernacer M . Motor conduction velocity and H‐reflex in infancy and childhood. II. ‐intra and extrauterine maturation of the nerve fibres. Development of the peripheral nerve from 1 month to 11 years of age. Electromyogr Clin Neurophysiol. 1978;18(1):11‐27. 679881

[41] Cruz Martinez A , Perez Conde ML , Ferrer MT . Motor conduction velocity and H‐reflex in infancy and childhood: I. Study in newborns, twins, and small‐for‐dates. Electromyogr Clin Neurophysiol. 1977;17(6):493‐505. 564771

[42] Kuczmarski RJ , Ogden CL , Guo SS , et al. 2000 CDC growth charts for the United States: methods and development. National Center for Health Statistics. Vital Health Stat. 2002;11:1. 12043359

[43] Wright MD , Poe MD , DeRenzo A , Haldal S , Escolar ML . Developmental outcomes of cord blood transplantation for Krabbe disease: a 15‐year study. Neurology. 2017;89(13):1365‐1372. 28855403 10.1212/WNL.0000000000004418 PMC5649761

[44] Wingard JR , Plotnick LP , Freemer CS , et al. Growth in children after bone marrow transplantation: busulfan plus cyclophosphamide versus cyclophosphamide plus total body irradiation. Blood. 1992;79(4):1068‐1073. 1737091

[45] Allewelt H , El‐Khorazaty J , Mendizabal A , et al. Late effects after umbilical cord blood transplantation in very young children after busulfan‐based, myeloablative conditioning. Biol Blood Marrow Transplant. 2016;22(9):1627‐1635. 27264632 10.1016/j.bbmt.2016.05.024

