# Correction to “Infantile Krabbe disease (0–12 months), progression, and recommended endpoints for clinical trials”

**DOI:** 10.1002/acn3.52275

**Published:** 2025-01-09

Article AID: ACN352114

**Table 1**

On Page 4, age at first evaluation for HSCT asymptomatic group should have a maximum of 40.7 not 40.1.

For initial psychosine levels, please update natural history median to 22.3 and range to 0.99–82.3, symptomatic HSCT median to 28.1 and range to 4.09–54.0, and asymptomatic HSCT range to 1.03–29.1.

For initial GALC levels, please update symptomatic HSCT median to 0.01 and asymptomatic HSCT range to 0.02–0.23.

**Table 2**

On Page 5, please update heading to say, “Summary of the growth percentiles in Natural History, Symptomatic HSCT, and Asymptomatic HSCT patients, indicating percentages below the third percentile for head circumference and below the fifth percentile for height, weight, and body mass index.”

**Sitting**

On Page 8, please update the last sentence from “52%” to “53%,” and replace “Table S6” with “Table S7” in the last sentence.

**Neurodevelopmental Function**

On Pages 10–11, the last sentence in the first paragraph of Page 11 (“*The Asympt HSCT group showed higher scores and greater gains over time than both the NH and Sympt HSCT groups for all domains (all p < 0.001) (Table* *S9* *)*.”) needs to be moved to Page 10 just after “*(Figs*. *S2* *and* *S3*, *Table* *S8* *)*.” and before “*The group performed slightly better…*.”

**Supporting Information**

Table S4

Table S4 is incorrectly listed as Table S5.

Tables [Link], [Link], and S9

Table S7 is incorrectly listed as Table S9. Table S8 is incorrectly listed as Table S7. Table S9 is incorrectly listed as Table S8.

Additionally, the tables and figures under Supporting Information do not have numbers or legends when downloaded. To address all issues, we have prepared new, reformatted PDFs with correct figure numbers and legends to be included in Supporting Information. Please just replace those files with the new ones provided. Thank you!

We apologize for these errors.

## Supporting information


**Figure S1.**.

**Figure S2.**.

**Figure S3.**.

**Table S1.**.

**Table S2.**.

**Table S3.**.

**Table S4.**.

**Table S5.**.

**Table S6.**.

**Table S7.**.

**Table S8.**.

**Table S9.**.

**Table S10a.**.

**Table S10b.**.

**Data S1.**.

