# Supplementary material for: Infantile Krabbe disease (0–12 months), progression, and recommended endpoints for clinical trials
Source: Ann Clin Transl Neurol. 2024 Nov 5;11(12):3064–80. doi: 10.1002/acn3.52114 (PMC11651195; doi:10.1002/acn3.52114)
Supplement: Supplementary file 1 — Data S1. [file ACN3-11-3064-s010.docx]

**Supplementary Text: Methodology Details**

**Inclusion and Exclusion Criteria**

Inclusion Criteria:

1. Diagnosis of infantile Krabbe disease, characterized by the following criteria outlined below:
   - Galactocerebrosidase (GALC) activity levels in leukocytes compatible with the diagnosis of Krabbe disease; and at least one of the following:
   - Elevated psychosine levels predictive of infantile disease onset by DBS; OR
   - CSF, MRI, NCV, or ABR consistent with Krabbe disease; OR
   - Two GALC mutations predictive to result in infantile onset phenotype.
2. Age at the time of symptom onset between birth and 12 months of age.

Exclusion criteria:

1. Presence of major congenital anomaly or any other condition that affects neurodevelopmental function.
2. Presence of any neurocognitive deficit or brain damage not attributable to Krabbe disease
3. Any other medical condition that would preclude participation in this study

**Informed Consent**

This research was approved by the IRBs of the University of North Carolina Chapel Hill (IRB #08-023, #07-1495) and the University of Pittsburgh (IRB-PRO11050036, PRO11050010). For study #08-023, UNC IRB granted a waiver of consent for patients evaluated prior to November 2006. Signed written consent was obtained for all patients who were evaluated after this date.

**Examination Schedule**

As stated in Martin et al. (2008) the recommended examination schedule was every 3-6 months for patients less than 1 year of age, 6-12 months for patients 2-3 years and once a year for patients older than 3 years^25^. However, family resources, including insurance approvals, as well as, patient illness resulted in missed or delayed evaluations.

**Examination Protocol**

The standardized protocol included an examination schedule in which the cognition and language was evaluated prior to motor with the physical and neurological examination occurring last. This minimized patient fatigue and encouraged their best performance on the neurodevelopmental scales. Any necessary blood draws were performed after the neurodevelopmental scales or were scheduled for a different day. An audiologic examination was performed prior to the developmental testing. If possible, MRIs and NCVs were scheduled to take place after the developmental testing and neurological examination, often on the following day. The protocol further specified that sedation could not be within 24 hours prior to the developmental testing.

A medical history and physical exam were done based on a neurodevelopmental protocol so that all caregivers were asked the same questions and all patients had the same baseline examination with additional items as needed. The medical history questionnaire was reviewed by expert physicians and nurse practitioners and then extracted by research assistants. Any questions by research assistants were clarified by the nurse practitioners or expert physicians. Physical exams were completed by neurodevelopmental pediatricians and trained nurse practitioners.

**Neurodevelopmental Measures**

Neurodevelopmental scales were administered by a combination of psychologists, speech pathologists, and physical therapists who were specifically trained to evaluate patient’s with Krabbe disease.

A detailed description of the measures used to evaluate these patients and a description of the standardized protocol is provided in Martin et al. (2008)^25^. It should be noted that in some cases changes were made over the years as scales were updated. Supplementary Table 2 provides the details of the scales used and the proportion of the evaluations performed using each scale.

**Table S2.** Neurodevelopmental measures used to assess each developmental domain.

| **Domain** | **Scales Used** | **# Evaluations** | **% of Evaluations** |
| --- | --- | --- | --- |
| **Cognitive** |  | 390 | 100% |
|  | Mullen Scales of Early Learning | 339 | 87% |
|  | Bayley Scales of Infant Development | 19 | 5% |
|  | The Capute Scales | 29 | 7% |
|  | Differential Ability Scales | 3 | <1% |
| **Adaptive** |  | 321 | 100% |
|  | Scales of Independent Behavior-Revised | 244 | 76% |
|  | Vineland III | 77 | 24% |
| **Receptive and Expressive Language** | | 349 | 100% |
|  | Mullen Scales of Early Learning | 339 | 97% |
|  | Preschool Language Scales 3 or 4 ed | 6 | 2% |
|  | Receptive-Expressive Emergent Language Test | 4 | 1% |
| **Gross Motor** |  |  |  |
|  | Peabody Developmental Motor Scales (PDMS) - 2 | 292 | 100% |
|  | Gross Motor Function Measure -88 | 237 | 100% |
| **Fine Motor** |  | 380 | 100% |
|  | Mullen Scales of Early Learning | 339 | 89% |
|  | The Capute Scales | 39 | 10% |
|  | Beery-Buktenica Developmental Test of Visual-Motor Integration | 2 | <1% |

Age equivalent scores were primarily used for analysis of developmental trajectories over time as presented in Martin et al. (2008)^25^. However, the PDMS-2 was also analyzed using the Quotient scores (standard score for PDMS-2) in order to make more specific inferences about the performance of the Asympt HSCT patients post-transplant development. The GMFM-88 was analyzed using the Total Score as age equivalent scores are not available for this scale. A subgroup of patients who were at risk for, but had not developed Infantile Krabbe disease, were used as a comparison group.

**Data Abstraction and Storage**

A Microsoft Access database was used to store psychometric measures and clinical data since 2003. Most of the data was extracted from the Access database to spreadsheets for analysis. Data was extracted from the Access database using SAS 9.4. Detailed chart review was performed by trained research assistants to add data about any variables that had not been previously included or were recoded to enhance the preciseness of the measure.
