# Supplementary material for: Infantile Krabbe disease (0–12 months), progression, and recommended endpoints for clinical trials
Source: Ann Clin Transl Neurol. 2024 Nov 5;11(12):3064–80. doi: 10.1002/acn3.52114 (PMC11651195; doi:10.1002/acn3.52114)
Supplement: Supplementary file 2 — Figure S1. [file ACN3-11-3064-s012.pdf]

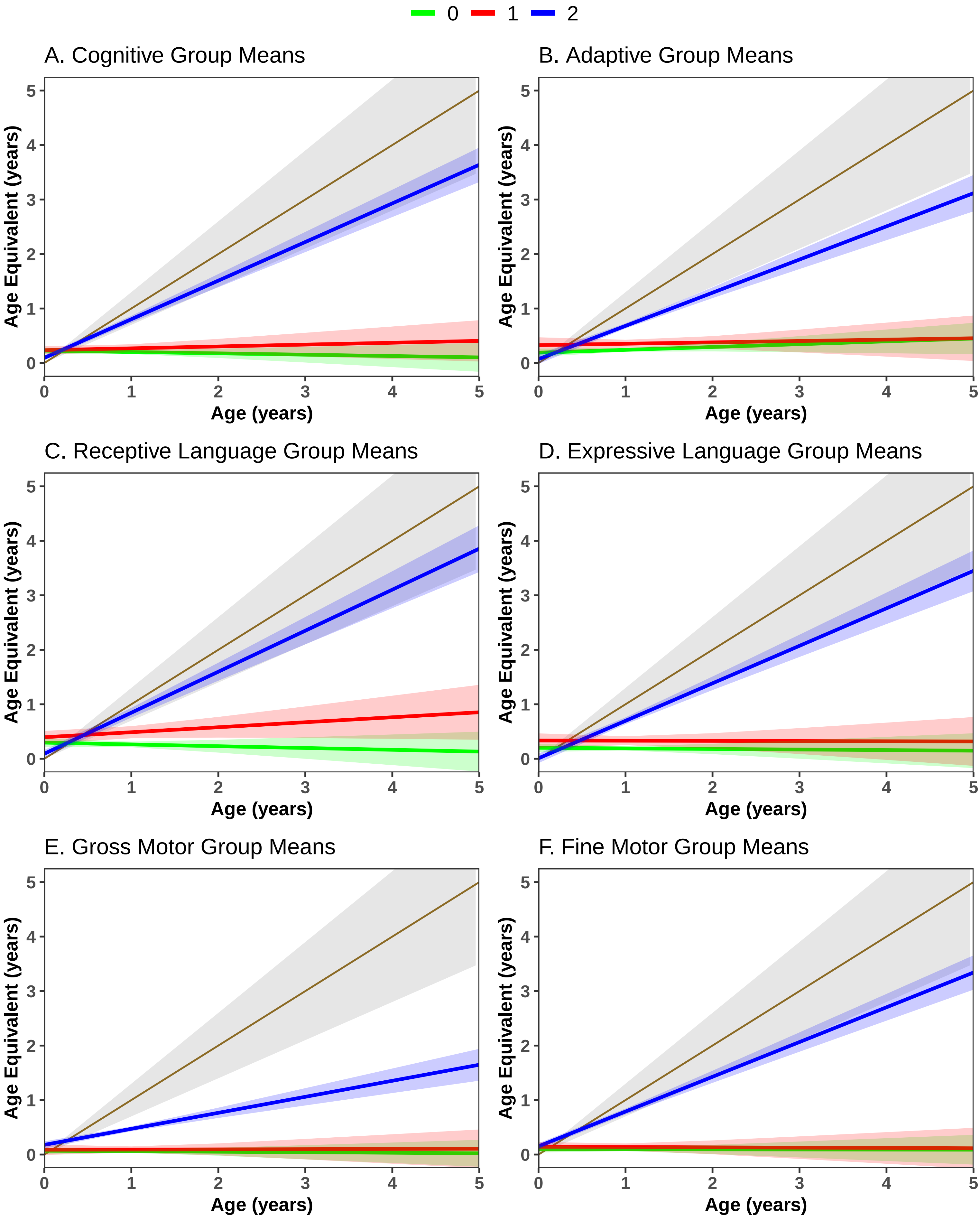

0
1
2
A. Cognitive Group Means
B. Adaptive Group Means
C. Receptive Language Group Means
D. Expressive Language Group Means
E. Gross Motor Group Means
F. Fine Motor Group Means
Age Equivalent (years)
Age (years)
0
1
2
3
4
5
