# Supplementary figures and images for: Infantile Krabbe disease (0–12 months), progression, and recommended endpoints for clinical trials
Source: Ann Clin Transl Neurol. 2024 Nov 5;11(12):3064–80. doi: 10.1002/acn3.52114 (PMC11651195; doi:10.1002/acn3.52114)

# PDMS Gross Motor Quotient

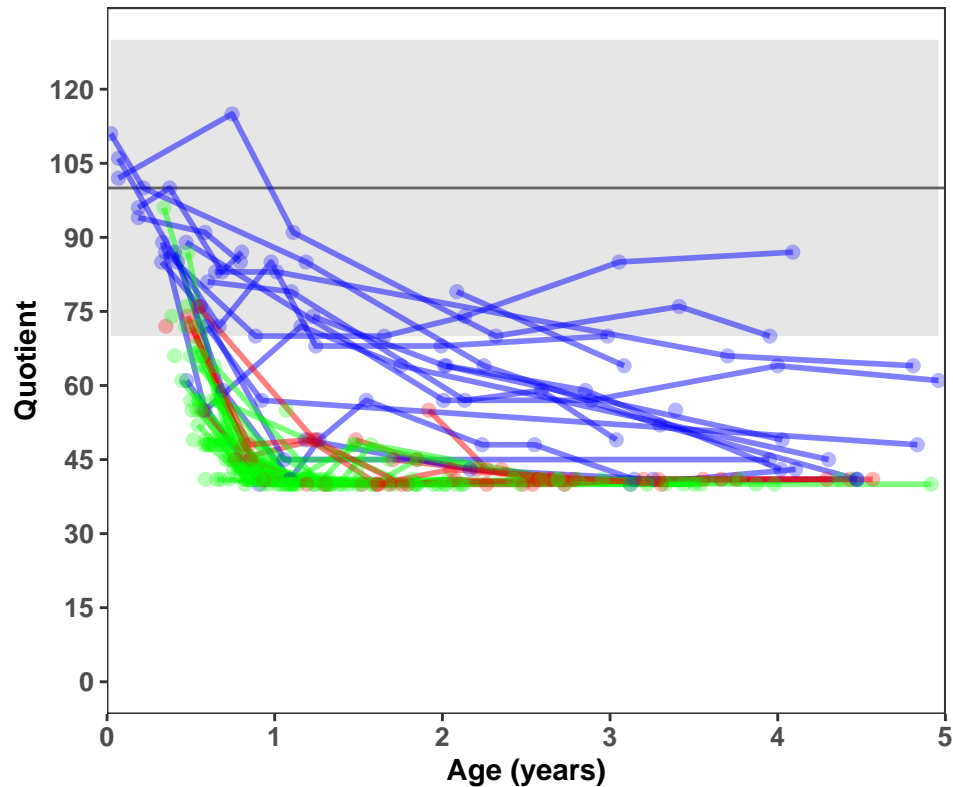

—●— NH —●— Symptomatic HSCT —●— Asymptomatic HSCT

Supplement: Supplementary file 3 — Figure S2. [file ACN3-11-3064-s007.pdf]

PDMS Gross Motor Quotient:  
Asymptomatic HSCT Mean

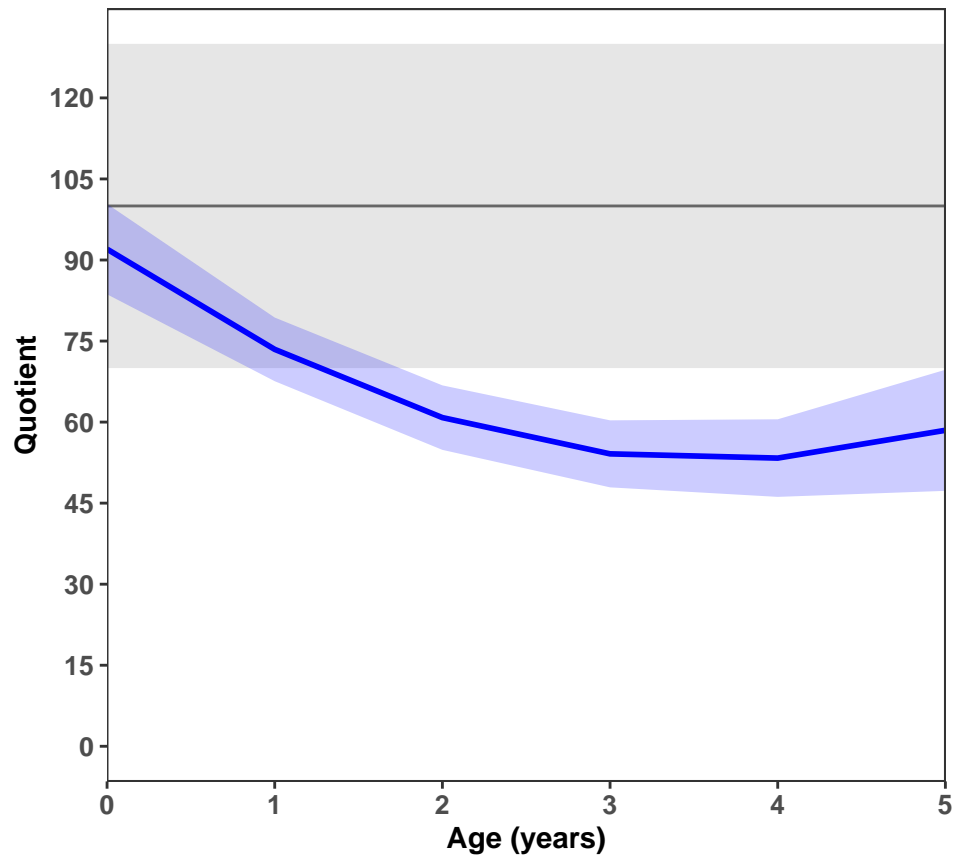

Supplement: Supplementary file 4 — Figure S3. [file ACN3-11-3064-s014.pdf]
