# Supplementary material for: Infantile Krabbe disease (0–12 months), progression, and recommended endpoints for clinical trials
Source: Ann Clin Transl Neurol. 2024 Nov 5;11(12):3064–80. doi: 10.1002/acn3.52114 (PMC11651195; doi:10.1002/acn3.52114)
Supplement: Supplementary file 5 — Table S1. [file ACN3-11-3064-s005.docx]

| **ID** | **Group** | **Allele 1** | **Allele 2** |
| --- | --- | --- | --- |
| 6 | Natural History | p.Arg168Cys_g.30kb deletion | p.Ile546Thr_c.1786+2insT |
| 9 | Natural History | p.Y158Lfsx3_p.Q312Q | P.G90R_p.Q312Q |
| 12 | Natural History | p.A21P_p.G111D_p.D248N | p.R184C_g.30kb deletion |
| 13 | Natural History | p.G36Afsx20 | p.G36Afsx20 |
| 17 | Natural History | 30kb deletion | c.592G>A (p.E198K) |
| 18 | Natural History | p.Pro148Thr | p.Arg168Cys_g.30Kbdeletion |
| 21 | Natural History | p.R168C_p.I546T | g.7.4Kb del_p.I546T |
| 25 | Natural History | c.1586C>T (p.Thr529Met) | c.1586C>T (p.Thr529Met) |
| 26 | Natural History | 24kb Deletion |  |
| 27 | Natural History | c.1714delinsAGC (p.Asp572Se | c.1714delinsAGC (p.Asp572Serfs*12) |
| 28 | Natural History | c.1472delA | c.967G>T (p.G323W) |
| 29 | Natural History | p.G284S_p.S434S_p.I546T | p.S434S_p.I546T_p.L634P |
| 37 | Natural History | c.195+1G>A | 30kb deletion |
| 40 | Natural History | p.R168C_30kb deletion | p.R168C_30kb deletion |
| 41 | Natural History | c.1541T>C (p.Phe514Ser) | c.909-10A>C |
| 44 | Natural History | 30kb deletion | 30kb deletion |
| 46 | Natural History | p.A5P-G9G_p.D232N_p.T513M | p.A5P-G9G_p.D232N_p.T513M |
| 49 | Natural History | p.D94D_p.E499K_p.I546T | p.R168C_g.30kb Del |
| 50 | Natural History | p.Ile546The_c.1766dupA (p.T | p.Ile546The_c.1766dupA (p.Tyr589fs*1) |
| 52 | Natural History | Exon 11-17 deletion |  |
| 54 | Natural History | p.N279I_p.S434S_p.I546T | p.N279I_p.S434S_p.I546T |
| 55 | Natural History | g.30kb deletion | c.1158del10 (p.Met387PhefsX?) |
| 60 | Natural History | c.242_243dupAG | c.1700A>C (p.Y567S) |
| 63 | Natural History | p.D94D_p.S434S_p.I546T_p.X6 | p.I128Lfsx27_p.R168C_p.I546T |
| 65 | Natural History | 14q31.3-14q32.12 |  |
| 66 | Natural History | p.R168C_g.30kb deletion | p.R168C_g.30kb deletion |
| 69 | Natural History | c.387C>G (p.Y129X) | c.1814dupA (p.Y605X) |
| 71 | Natural History | Exon 11-17 deletion | c.379C>T (p.R127X) |
| 73 | Natural History | c.749T>C (p.I250T) | c.1586C>T (p.T529M) |
| 74 | Natural History | p.Arg168Cys_30kb deletion | p.Gly41Ser_p.Ser343Ser_p.Ile546Thr |
| 77 | Natural History | Exon 11-17 deletion | Exon 11-17 deletion |
| 82 | Natural History | p.R168C_g.30kb del | p.Y319C_p.S434S_p.I546T |
| 83 | Natural History | c.1186C>T (p.Arg396Trp) | Exon 11-17 deletion |
| 84 | Natural History | p.P239H_p.I546T | p.S434S_p.V320M_p.R515C_p.I546T |
| 86 | Natural History | 30kb deletion | c.868C>T (p.R290C) |
| 87 | Natural History | c.597G>T (p.Arg199Ser) | c.1161+6532_polyA+9kbdel |
| 96 | Natural History | Exon 11-17 deletion | Exon 11-17 deletion |
| 97 | Natural History | p.R168C_30 kb deletion | c.1637G>A (p.G553R)_c.1786+5 G>A (p.A625T) |
| 101 | Natural History | c.281delC (p.Ser94Phefs*5) | c.583-6T>A |
| 103 | Natural History | c.908C>T (p.Ser303Phe) | c.1685T>C (p.Ile562Thr) |
| 111 | Natural History | Exon 11-17 deletion | c.1793G>A (p.Trp598*) |
| 117 | Natural History | p.Ala5Pro_p.Gly9Gly_p.Arg19 | p.Ser434Ser_p.Ile546The_p.Leu616Pro |
| 119 | Natural History | c.1586C>T (p.T529M) | c.1700A>C (p.Y567S) |
| 121 | Natural History | p.R168C_30kb deletion | p.R168C_30kb deletion |
| 126 | Natural History | p.W147X_p.S434S_p.I546T | p.W147X_p.S434S_p.I546T |
| 127 | Natural History | c.926T>C (p.L309X) |  |
| 130 | Natural History | p.Ile546Thr_p.Tyr551Ser | c.1110_1119delCATGGTAAC (p.Met371Phefs) |
| 134 | Natural History | g.30kb deletion_p.Arg168Cys | g.30kb deletion_p.Arg168Cys |
| 136 | Natural History | p.R168C_g.30kb del | p.T513M_p.I546T |
| 137 | Natural History | c.1630 G>A (p.Asp544Asn) | c.1630 G>A (p.Asp544Asn) |
| 43 | HSCT Symptomatic | p.Arg168Cys_30kb deletion | c.572delA_p.Ile546Thr |
| 62 | HSCT Symptomatic | p.R168C_30kb deletion | p.Y319C_p.I546T |
| 95 | HSCT Symptomatic | c.388G>A (p.Glu130Lys) | c.388G>A (p.Glu130Lys) |
| 102 | HSCT Symptomatic | p.A5P-G9G_p.D232N_p.A290T | p.R168C_g.30kb del |
| 16 | HSCT Asymptomatic | del30kb_R168C | c.-335G>A_p.G360Dfs*2# |
| 53 | HSCT Asymptomatic | c.622-1G>T | Exon 11-17 deletion |
| 58 | HSCT Asymptomatic | c.599C>A (p.Ser200Ter) | c.956A>G (p.Tyr319Cys) |
| 59 | HSCT Asymptomatic | p.W162X_p.S434Sp.I546T | p.Y474N_p.S434Sp.I546T |
| 61 | HSCT Asymptomatic | c.749T>C (p.I250T) | c.1171_1175het_delCATTCinsA |
| 78 | HSCT Asymptomatic | c.658C>T (p.Arg220Ter) | c.956A>G (pTyr319Cys) |
| 79 | HSCT Asymptomatic | 30kb deletion | 30kb deletion |
| 114 | HSCT Asymptomatic | c.658C>T (p.Arg220Ter) | c.956A>G (pTyr319Cys) |
| 122 | HSCT Asymptomatic | p.R168C_g.30kbdel | p.A209E |
