# Supplementary material for: Infantile Krabbe disease (0–12 months), progression, and recommended endpoints for clinical trials
Source: Ann Clin Transl Neurol. 2024 Nov 5;11(12):3064–80. doi: 10.1002/acn3.52114 (PMC11651195; doi:10.1002/acn3.52114)
Supplement: Supplementary file 6 — Table S3. [file ACN3-11-3064-s001.docx]

| **Race** | **Number of patients** |
| --- | --- |
| White | 123 |
| Black | 5 |
| Biracial | 2 |
| Asian | 1 |
