# Supplementary material for: Infantile Krabbe disease (0–12 months), progression, and recommended endpoints for clinical trials
Source: Ann Clin Transl Neurol. 2024 Nov 5;11(12):3064–80. doi: 10.1002/acn3.52114 (PMC11651195; doi:10.1002/acn3.52114)
Supplement: Supplementary file 7 — Table S4. [file ACN3-11-3064-s009.docx]

| **Complications** | **Number of patients (%)** |
| --- | --- |
| Delivered via C-section | 47 (34%) |
| Neonatal complications | 57 (42%) |
| Multiple concomitant complications | 17 (12%) |
