# Supplementary material for: Infantile Krabbe disease (0–12 months), progression, and recommended endpoints for clinical trials
Source: Ann Clin Transl Neurol. 2024 Nov 5;11(12):3064–80. doi: 10.1002/acn3.52114 (PMC11651195; doi:10.1002/acn3.52114)
Supplement: Supplementary file 8 — Table S5. [file ACN3-11-3064-s006.docx]

| **# of Evaluations** | **Natural History** | **HSCT Symptomatic** | **HSCT Asymptomatic** |
| --- | --- | --- | --- |
| **1** | 47 | 2 | 2 |
| **2** | 22 | 4 | 1 |
| **3** | 13 | 3 | 1 |
| **4** | 4 | 1 | 1 |
| **5** | 5 | 0 | 3 |
| **6** | 1 | 3 | 2 |
| **7** | 2 | 1 | 3 |
| **8** | 1 | 0 | 2 |
| **9** | 1 | 2 | 5 |
| **10** | 0 | 2 | 2 |
| **11** | 0 | 0 | 1 |
| **Total** | 96 | 18 | 23 |
