# Supplementary material for: Infantile Krabbe disease (0–12 months), progression, and recommended endpoints for clinical trials
Source: Ann Clin Transl Neurol. 2024 Nov 5;11(12):3064–80. doi: 10.1002/acn3.52114 (PMC11651195; doi:10.1002/acn3.52114)
Supplement: Supplementary file 9 — Table S6. [file ACN3-11-3064-s013.docx]

|  | 0-2 months | | 3-5 months | | 6-8 months | | 9-11 months | | 12-17 months | | 18-23 months | | 24-35 months | | 36-60 months | |
| --- | --- | --- | --- | --- | --- | --- | --- | --- | --- | --- | --- | --- | --- | --- | --- | --- |
|  | N | % | N | % | N | % | N | % | N | % | N | % | N | % | N | % |
| **Diarrhea** |  |  |  |  |  |  |  |  |  |  |  |  |  |  |  |  |
| NH | 0/1 | 0% | 3/14 | 22% | 3/37 | 8% | 1/29 | 3% | 0/35 | 0% | 0/18 | 0% | 3/16 | 19% | 0/10 | 0% |
| Sympt | - | - | 2/3 | 68% | 0/8 | 0% | 1/4 | 25% | 1/7 | 14% | 0/9 | 0% | 0/8 | 0% | 0/8 | 0% |
| Asympt | 0/20 | 0% | 0/8 | 0% | 2/10 | 20% | 0/7 | 0% | 1/13 | 8% | 0/9 | 0% | 1/13 | 8% | 0/19 | 0% |
| **Constipation** |  |  |  |  |  |  |  |  |  |  |  |  |  |  |  |  |
| NH | 0/1 | 0% | 4/15 | 27% | 17/41 | 41% | 19/32 | 59% | 25/38 | 66% | 13/20 | 65% | 11/17 | 65% | 10/11 | 91% |
| Sympt | - | - | 0/3 | 0% | 3/8 | 38% | 1/4 | 25% | 1/7 | 14% | 0/9 | 0% | 2/8 | 25% | 1/8 | 13% |
| Asympt | 0/19 | 0% | 0/8 | 0% | 0/10 | 0% | 0/7 | 0% | 0/13 | 0% | 0/9 | 0% | 3/13 | 23% | 3/19 | 16% |
| **Feeding Difficulties** | |  |  |  |  |  |  |  |  |  |  |  |  |  |  |  |
| NH | 1/1 | 100% | 13/15 | 87% | 41/43 | 95% | 32/33 | 97% | 38/40 | 95% | 19/19 | 100% | 15/16 | 94% | 10/10 | 100% |
| Sympt | - | - | 3/6 | 50% | 8/9 | 89% | 4/4 | 100% | 6/7 | 86% | 10/10 | 100% | 7/8 | 88% | 8/8 | 100% |
| Asympt | 1/20 | 5% | 4/8 | 50% | 4/10 | 40% | 2/7 | 29% | 3/13 | 23% | 3/9 | 33% | 8/13 | 62% | 8/19 | 42% |
| **Reflux** |  |  |  |  |  |  |  |  |  |  |  |  |  |  |  |  |
| NH | 1/1 | 100% | 13/16 | 81% | 30/37 | 81% | 18/30 | 60% | 17/34 | 50% | 9/19 | 47% | 4/16 | 25% | 7/10 | 70% |
| Sympt | - | - | 2/6 | 33% | 4/9 | 44% | 2/4 | 50% | 1/7 | 14% | 6/10 | 60% | 2/8 | 25% | 4/8 | 50% |
| Asympt | 1/19 | 5% | 0/8 | 0% | 0/10 | 0% | 0/7 | 0% | 1/13 | 8% | 1/9 | 11% | 0/13 | 0% | 2/19 | 11% |
| **Swallowing Difficulties** | | |  |  |  |  |  |  |  |  |  |  |  |  |  |  |
| NH | 0/1 | 0% | 11/14 | 79% | 39/42 | 93% | 28/32 | 88% | 37/37 | 100% | 18/18 | 100% | 15/15 | 100% | 10/10 | 100% |
| Sympt | - | - | 3/6 | 50% | 8/9 | 89% | 4/4 | 100% | 6/7 | 86% | 8/8 | 100% | 8/8 | 100% | 8/8 | 100% |
| Asympt | 2/20 | 10% | 2/8 | 25% | 2/10 | 20% | 0/6 | 0% | 1/12 | 8% | 0/8 | 0% | 4/11 | 36% | 3/17 | 18% |
| **Sialorrhea with need of Suctioning** | |  |  |  |  |  |  |  |  |  |  |  |  |  |  |  |
| NH | 0/1 | 0% | 1/13 | 8% | 10/39 | 26% | 13/32 | 41% | 22/40 | 55% | 17/20 | 85% | 14/17 | 82% | 7/11 | 65% |
| Sympt | - |  | 0/4 | 0% | 1/9 | 11% | 1/4 | 25% | 1/7 | 14% | 3/9 | 33% | 4/8 | 50% | 5/8 | 63% |
| Asympt | 0/20 | 0% | 0/8 | 0% | 0/10 | 0% | 0/7 | 0% | 0/13 | 0% | 0/9 | 0% | 0/13 | 0% | 1/19 | 5% |
| **Head Control** |  |  |  |  |  |  |  |  |  |  |  |  |  |  |  |  |
| NH | 0/1 | 0% | 2/14 | 14% | 7/44 | 16% | 7/34 | 21% | 8/41 | 20% | 0/20 | 0% | 1/17 | 6% | 1/11 | 9% |
| Sympt | - | - | 2/5 | 40% | 3/8 | 38% | 2/4 | 50% | 2/7 | 29% | 5/10 | 50% | 4/8 | 50% | 4/8 | 50% |
| Asympt | 5/18 | 28% | 3/9 | 33% | 3/10 | 30% | 1/7 | 15% | 4/12 | 33% | 3/9 | 33% | 5/13 | 38% | 9/19 | 47% |
| **Sit Unassisted** |  |  |  |  |  |  |  |  |  |  |  |  |  |  |  |  |
| NH | NA | NA | NA | NA | 0/41 | 0% | 0/34 | 0% | 0/41 | 0% | 0/20 | 0% | 0/17 | 0% | 0/11 | 0% |
| Sympt | NA | NA | NA | NA | 0/9 | 0% | 0/4 | 0% | 0/7 | 0% | 0/10 | 0% | 0/8 | 0% | 0/8 | 0% |
| Asympt | NA | NA | NA | NA | 0/10 | 0% | 2/6 | 33% | 7/13 | 54% | 7/9 | 78% | 12/13 | 92% | 14/18 | 78% |
| **Axial Hypotonia** | |  |  |  |  |  |  |  |  |  |  |  |  |  |  |  |
| NH | 1/1 | 100% | 6/14 | 43% | 32/40 | 80% | 32/34 | 94% | 39/41 | 95% | 20/20 | 100% | 16/16 | 100% | 11/11 | 100% |
| Sympt | - | - | 2/3 | 67% | 6/9 | 67% | 2/4 | 50% | 6/7 | 86% | 9/9 | 100% | 8/8 | 100% | 8/8 | 100% |
| Asympt | 2/20 | 10% | 4/8 | 50% | 5/10 | 50% | 5/7 | 71% | 9/12 | 75% | 8/9 | 89% | 11/13 | 85% | 15/19 | 79% |
| **Appendicular Spasticity** | | |  |  |  |  |  |  |  |  |  |  |  |  |  |  |
| NH | 1/1 | 100% | 13/13 | 100% | 42/44 | 95% | 31/33 | 94% | 34/41 | 83% | 14/20 | 70% | 10/17 | 59% | 8/11 | 73% |
| Sympt | - | - | 6/6 | 100% | 7/9 | 78% | 5/5 | 100% | 7/7 | 100% | 8/8 | 100% | 8/8 | 100% | 8/8 | 100% |
| Asympt | 3/20 | 15% | 2/8 | 25% | 4/10 | 40% | 3/7 | 43% | 7/11 | 64% | 5/9 | 56% | 9/13 | 69% | 14/19 | 74% |
| **Clasped Thumb** | |  |  |  |  |  |  |  |  |  |  |  |  |  |  |  |
| NH | 1/1 | 100% | 12/15 | 80% | 38/44 | 86% | 27/33 | 82% | 32/40 | 80% | 14/20 | 70% | 12/17 | 71% | 3/10 | 30% |
| Sympt | - | - | 6/6 | 100% | 7/8 | 88% | 4/5 | 80% | 5/7 | 71% | 4/9 | 44% | 4/8 | 50% | 5/8 | 63% |
| Asympt | 2/20 | 10% | 3/9 | 33% | 3/9 | 33% | 2/7 | 29% | 2/13 | 16% | 1/9 | 11% | 213 | 15% | 4/19 | 21% |
| **Absent or Abnormal Deep Tendon Reflexes** | | | | |  |  |  |  |  |  |  |  |  |  |  |  |
| NH | 1/1 | 100% | 11/13 | 85% | 39/42 | 93% | 34/34 | 100% | 41/41 | 100% | 19/20 | 95% | 17/17 | 100% | 10/10 | 100% |
| Sympt | - | - | 5/6 | 83% | 8/9 | 89% | 5/5 | 100% | 6/7 | 86% | 9/10 | 90% | 8/8 | 100% | 8/8 | 100% |
| Asympt | 4/19 | 21% | 3/8 | 38% | 5/10 | 50% | 3/7 | 43% | 10/12 | 84% | 5/8 | 63% | 10/12 | 83% | 16/18 | 89% |
| **Bulging Fontanelle** | |  |  |  |  |  |  |  |  |  |  |  |  |  |  |  |
| NH | 0/1 | 0% | 4/15 | 27% | 10/41 | 24% | 7/33 | 21% | 14/40 | 35% | 9/20 | 45% | 8/17 | 48% | 5/10 | 50% |
| Sympt | - | - | 1/6 | 17% | 1/9 | 20% | 2/4 | 50% | 2/7 | 29% | 2/10 | 20% | 2/8 | 25% | 0/8 | 0% |
| Asympt | 4/20 | 20% | 4/7 | 57% | 6/10 | 60% | 4/7 | 57% | 7/13 | 54% | 4/8 | 50% | 1/13 | 8% | 1/18 | 6% |
| **Staring Episodes** | |  |  |  |  |  |  |  |  |  |  |  |  |  |  |  |
| NH | 0/1 | 0% | 4/16 | 25% | 17/44 | 39% | 19/34 | 56% | 20/41 | 49% | 17/20 | 85% | 14/17 | 83% | 10/11 | 90% |
| Sympt | - | - | 1/6 | 17% | 2/9 | 22% | 2/5 | 40% | 1/7 | 14% | 3/11 | 27% | 4/8 | 50% | 3/8 | 38% |
| Asympt | 0/20 | 0% | 0/10 | 0% | 0/13 | 0% | 1/8 | 13% | 1/13 | 8% | 0/9 | 0% | 1/16 | 6% | 0/20 | 0% |
| **Clinical Seizures** | |  |  |  |  |  |  |  |  |  |  |  |  |  |  |  |
| NH | 0/1 | 0% | 1/15 | 7% | 4/40 | 10% | 6/33 | 18% | 10/38 | 26% | 6/19 | 32% | 4/17 | 24% | 4/11 | 36% |
| Sympt | - | - | 0/1 | 0% | 1/9 | 11% | 1/4 | 25% | 0/7 | 0% | 2/9 | 22% | 1/8 | 13% | 2/8 | 25% |
| Asympt | 0/19 | 0% | 0/8 | 0% | 0/10 | 0% | 0/7 | 0% | 0/13 | 0% | 1/9 | 11% | 2/13 | 15% | 2/19 | 11% |
| **Hip Asymmetry** | |  |  |  |  |  |  |  |  |  |  |  |  |  |  |  |
| NH | 0/1 | 0% | 0/11 | 0% | 6/38 | 16% | 9/33 | 27% | 17/41 | 41% | 8/20 | 40% | 12/17 | 71% | 11/11 | 100% |
| Sympt | - | - | 0/2 | 0% | 1/9 | 11% | 2/4 | 50% | 2/7 | 29% | 4/10 | 40% | 3/8 | 38% | 3/8 | 38% |
| Asympt | 0/20 | 0% | 0/8 | 0% | 1/10 | 10% | 0/7 | 0% | 0/13 | 0% | 0/9 | 0% | 1/13 | 8% | 3/17 | 18% |
| **Scoliosis** |  |  |  |  |  |  |  |  |  |  |  |  |  |  |  |  |
| NH | 0/1 | 0% | 0/13 | 0% | 6/40 | 15% | 8/33 | 24% | 18/39 | 46% | 10/20 | 50% | 10/17 | 59% | 11/11 | 100% |
| Sympt | - | - | 0/2 | 0% | 0/9 | 0% | 0/4 | 0% | 0/7 | 0% | 2/9 | 22% | 7/8 | 88% | 5/8 | 63% |
| Asympt | 0/20 | 0% | 0/8 | 0% | 1/10 | 10% | 0/7 | 0% | 0/13 | 0% | 2/9 | 22% | 2/13 | 15% | 3/19 | 16% |
|  |  |  |  |  |  |  |  |  |  |  |  |  |  |  |  |  |
| **Vision and Hearing** | |  |  |  |  |  |  |  |  |  |  |  |  |  |  |  |
| **Visual Tracking Difficulties** |  |  |  |  |  |  |  |  |  |  |  |  |  |  |  |  |
| NH | 0/1 | 0% | 12/16 | 75% | 28/43 | 65% | 20/33 | 61% | 32/41 | 78% | 15/20 | 75% | 15/17 | 88% | 9/11 | 82% |
| Sympt | - | - | 3/6 | 50% | 4/9 | 44% | 2/5 | 40% | 2/7 | 29% | 3/10 | 30% | 4/8 | 50% | 3/8 | 38% |
| Asympt | 1/20 | 5% | 2/9 | 22% | 1/10 | 10% | 0/7 | 0% | 0/13 | 0% | 0/9 | 0% | 0/13 | 0% | 0/19 | 0% |
| **VEP** |  |  |  |  |  |  |  |  |  |  |  |  |  |  |  |  |
| NH | - | - | 2/6 | 33% | 3/17 | 18% | 4/16 | 25% | 7/13 | 53% | 3/8 | 38% | - | - | 2/2 | 100% |
| Sympt | - | - | 0/1 | 0% | 1/1 | 100% | - | - | 3/5 | 60% | 2/5 | 40% | 3/6 | 50% | 0/5 | 0% |
| Asympt | 5/8 | 63% | 0/3 | 0% | 0/4 | 0% | 0/5 | 0% | 2/6 | 33% | 1/2 | 50% | 1/3 | 33% | 0/4 | 0% |
| **ABR** |  |  |  |  |  |  |  |  |  |  |  |  |  |  |  |  |
| NH | - | - | 3/5 | 60% | 17/18 | 95% | 12/13 | 92% | 10/11 | 91% | 6/6 | 100% | 2/3 | 67% | 4/4 | 100% |
| Sympt | - | - | 1/1 | 100% | 6/6 | 100% | 0/1 | 0% | 6/6 | 100% | 7/7 | 100% | 6/8 | 75% | 2/4 | 50% |
| Asympt | 8/11 | 73% | 4/4 | 100% | 3/5 | 60% | 2/3 | 67% | 4/6 | 67% | 2/3 | 67% | 4/5 | 80% | 5/6 | 84% |
