# Supplementary material for: Infantile Krabbe disease (0–12 months), progression, and recommended endpoints for clinical trials
Source: Ann Clin Transl Neurol. 2024 Nov 5;11(12):3064–80. doi: 10.1002/acn3.52114 (PMC11651195; doi:10.1002/acn3.52114)
Supplement: Supplementary file 10 — Table S7. [file ACN3-11-3064-s003.docx]

|  | **Cognitive** | | | **Adaptive** | | | **Receptive Language** | | | **Expressive Language** | | | **Gross Motor** | | | **Fine Motor** | | |
| --- | --- | --- | --- | --- | --- | --- | --- | --- | --- | --- | --- | --- | --- | --- | --- | --- | --- | --- |
| **Variable** | **β** | **SE** | **p** | **β** | **SE** | **p** | **β** | **SE** | **p** | **β** | **SE** | **p** | **β** | **SE** | **p** | **β** | **SE** | **p** |
| Intercept | 0.16 | 0.06 | 0.005 | 0.32 | 0.04 | <0.001 | 0.21 | 0.08 | 0.008 | 0.17 | 0.07 | 0.009 | 0.05 | 0.05 | 0.357 | 0.09 | 0.06 | 0.105 |
| Age (years) | -0.02 | 0.03 | 0.417 | 0.05 | 0.03 | 0.063 | -0.03 | 0.04 | 0.441 | -0.01 | 0.04 | 0.785 | -0.01 | 0.03 | 0.754 | 0.00 | 0.03 | 0.950 |
| Group |  |  | <0.001 |  |  | <0.001 |  |  | <0.001 |  |  | <0.001 |  |  | <0.001 |  |  | <0.001 |
| HSCT Asymptomatic | 1.70 | 0.10 | <0.001 | 1.25 | 0.05 | <0.001 | 1.76 | 0.13 | <0.001 | 1.55 | 0.11 | <0.001 | 0.87 | 0.08 | <0.001 | 1.65 | 0.09 | <0.001 |
| HSCT Symptomatic | 0.16 | 0.11 | 0.146 | 0.08 | 0.06 | 0.169 | 0.41 | 0.14 | 0.006 | 0.15 | 0.11 | 0.190 | 0.05 | 0.09 | 0.578 | 0.04 | 0.10 | 0.725 |
| Natural History | 0.00 |  |  | 0.00 |  |  | 0.00 |  |  | 0.00 |  |  | 0.00 |  |  | 0.00 |  |  |
| Group x Age |  |  | <0.001 |  |  | <0.001 |  |  | <0.001 |  |  | <0.001 |  |  | <0.001 |  |  | <0.001 |
| HSCT Asymptomatic | 0.73 | 0.05 | <0.001 | 0.54 | 0.04 | <0.001 | 0.79 | 0.06 | <0.001 | 0.70 | 0.06 | <0.001 | 0.30 | 0.04 | <0.001 | 0.64 | 0.05 | <0.001 |
| HSCT Symptomatic | 0.06 | 0.05 | 0.254 | -0.04 | 0.05 | 0.420 | 0.12 | 0.07 | 0.079 | 0.01 | 0.07 | 0.914 | 0.01 | 0.05 | 0.806 | 0.00 | 0.05 | 0.947 |
| Natural History | 0.00 |  |  | 0.00 |  |  | 0.00 |  |  | 0.00 |  |  | 0.00 |  |  | 0.00 |  |  |
| **Estimates** | **Diff** | **SE** | **p** | **Diff** | **SE** | **p** | **Diff** | **SE** | **p** | **Diff** | **SE** | **p** | **Diff** | **SE** | **p** | **Diff** | **SE** | **p** |
| Asympt vs Sympt @ 2.5 years | 1.55 | 0.12 | <0.001 | 1.17 | 0.05 | <0.001 | 1.35 | 0.16 | <0.001 | 1.40 | 0.12 | <0.001 | 0.82 | 0.10 | <0.001 | 1.62 | 0.11 | <0.001 |
| Asympt vs Sympt @ 4.5 years | 2.90 | 0.22 | <0.001 | 2.34 | 0.13 | <0.001 | 2.67 | 0.30 | <0.001 | 2.79 | 0.26 | <0.001 | 1.40 | 0.20 | <0.001 | 2.90 | 0.22 | <0.001 |
| Asympt vs NH @ 2.5 years | 1.70 | 0.10 | <0.001 | 1.25 | 0.05 | <0.001 | 1.76 | 0.13 | <0.001 | 1.55 | 0.11 | <0.001 | 0.87 | 0.08 | <0.001 | 1.65 | 0.09 | <0.001 |
| Asympt vs NH @ 4.5 years | 3.17 | 0.18 | <0.001 | 2.34 | 0.11 | <0.001 | 3.33 | 0.25 | <0.001 | 2.95 | 0.22 | <0.001 | 1.47 | 0.17 | <0.001 | 2.93 | 0.18 | <0.001 |
| Sympt vs NH @ 2.5 years | 0.16 | 0.11 | 0.146 | 0.08 | 0.06 | 0.169 | 0.41 | 0.14 | 0.006 | 0.15 | 0.11 | 0.190 | 0.05 | 0.09 | 0.578 | 0.04 | 0.10 | 0.725 |
| Sympt vs NH @ 4.5 years | 0.27 | 0.21 | 0.187 | 0.00 | 0.14 | 0.996 | 0.66 | 0.28 | 0.020 | 0.17 | 0.24 | 0.495 | 0.08 | 0.19 | 0.692 | 0.03 | 0.21 | 0.890 |
