# Supplementary material for: Infantile Krabbe disease (0–12 months), progression, and recommended endpoints for clinical trials
Source: Ann Clin Transl Neurol. 2024 Nov 5;11(12):3064–80. doi: 10.1002/acn3.52114 (PMC11651195; doi:10.1002/acn3.52114)
Supplement: Supplementary file 11 — Table S8. [file ACN3-11-3064-s011.docx]

|  | **PDMS Quotient** | | |
| --- | --- | --- | --- |
| **Variable** | **B** | **SE** | **p** |
| Intercept | 56.73 | 3.05 | <0.001 |
| Age (centered at 2.5 years) | -6.71 | 1.16 | <0.000 |
| Age | 2.96 | 0.67 | <0.001 |
|  |  |  |  |
| **Estimates** | **B** | **SE** | **p** |
| Asymptomatic HSCT @ 0 years | 92.01 | 4.18 | <0.001 |
| Asymptomatic HSCT @ 1 years | 73.46 | 2.95 | <0.001 |
| Asymptomatic HSCT @ 2 years | 60.83 | 2.98 | <0.001 |
| Asymptomatic HSCT @ 3 years | 54.12 | 3.10 | <0.001 |
| Asymptomatic HSCT @ 4 years | 53.34 | 3.59 | <0.001 |
| Asymptomatic HSCT @ 5 years | 58.48 | 5.60 | <0.001 |
|  |  |  |  |
