# Supplementary material for: Infantile Krabbe disease (0–12 months), progression, and recommended endpoints for clinical trials
Source: Ann Clin Transl Neurol. 2024 Nov 5;11(12):3064–80. doi: 10.1002/acn3.52114 (PMC11651195; doi:10.1002/acn3.52114)
Supplement: Supplementary file 12 — Table S9. [file ACN3-11-3064-s002.docx]

| **Sitting independently** | | |
| --- | --- | --- |
| **Group** | **12 months** | **24 months** |
| Natural History | 6% | 8% |
| Symptomatic HSCT | 0% | 0% |
| Asymptomatic HSCT | 42% | 53% |
| **Walking independently** | | |
| **Group** | **24 months** | **48 months** |
| Natural History | 0% | 0% |
| Symptomatic HSCT | 0% | 0% |
| Asymptomatic HSCT | 10% | 21% |
