# Supplementary material for: Infantile Krabbe disease (0–12 months), progression, and recommended endpoints for clinical trials
Source: Ann Clin Transl Neurol. 2024 Nov 5;11(12):3064–80. doi: 10.1002/acn3.52114 (PMC11651195; doi:10.1002/acn3.52114)
Supplement: Supplementary file 13 — Table S10a. [file ACN3-11-3064-s008.docx]

|  | **Galactocerebrosidase** | | | | | |
| --- | --- | --- | --- | --- | --- | --- |
| **Age (months)** | **N** | **Median** | **Mean** | **SD** | **Min** | **Max** |
| 0-36 | 101 | 0.1 | 0.1 | 0.1 | 0.0 | 1.1 |
|  |  |  |  |  |  |  |
|  | **Psychosine** | | | | | |
| **Age (months)** | **N** | **Median** | **Mean** | **SD** | **Min** | **Max** |
| 0-3 | 27 | 11.2 | 20.0 | 21.2 | 1.0 | 76.9 |
| 3-6 | 10 | 38.4 | 36.1 | 15.4 | 14.8 | 54.0 |
| 6-9 | 43 | 28.3 | 31.5 | 16.8 | 5.9 | 82.3 |
| 9-12 | 13 | 16.6 | 18.4 | 10.8 | 5.1 | 36.4 |
| 12-18 | 18 | 17.9 | 19.7 | 16.0 | 1.0 | 60.2 |
| 18-24 | 10 | 12.0 | 14.3 | 9.2 | 4.0 | 36.5 |
| 24-36 | 9 | 9.1 | 12.4 | 9.3 | 2.9 | 33.0 |
| 36-60 | 8 | **8** | 8 | 4 | 4 | 14 |
|  |  |  |  |  |  |  |
|  | **CSF Protein** | | | | | |
| **Age (months)** | **N** | **Median** | **Mean** | **SD** | **Min** | **Max** |
| 0-3 | 13 | 239 | 264 | 154 | 89 | 547 |
| 3-6 | 10 | 250 | 241 | 141 | 39 | 571 |
| 6-9 | 39 | 255 | 236 | 87 | 43 | 387 |
| 9-12 | 13 | 165 | 212 | 157 | 60 | 594 |
| 12-18 | 17 | 208 | 201 | 88 | 51 | 339 |
| 18-24 | 10 | 203 | 191 | 114 | 34 | 375 |
| 24-36 | 8 | 282 | 265 | 133 | 61 | 405 |
| 36-60 | 3 | 135 | 116 | 33 | 77 | 135 |
|  |  |  |  |  |  |  |
