# Supplementary material for: Infantile Krabbe disease (0–12 months), progression, and recommended endpoints for clinical trials
Source: Ann Clin Transl Neurol. 2024 Nov 5;11(12):3064–80. doi: 10.1002/acn3.52114 (PMC11651195; doi:10.1002/acn3.52114)
Supplement: Supplementary file 14 — Table S10b. [file ACN3-11-3064-s004.docx]

|  | **Galactocerebrosidase** | | | | | | | | | | | | |
| --- | --- | --- | --- | --- | --- | --- | --- | --- | --- | --- | --- | --- | --- |
|  | **HSCT Symptomatic** | | | | | |  | **HSCT Asymptomatic** | | | | | |
| **Months from HSCT** | **N** | **Median** | **Mean** | **SD** | **Min** | **Max** |  | **N** | **Median** | **Mean** | **SD** | **Min** | **Max** |
| 0-3 | 4 | 3.45 | 3.25 | 0.44 | 2.60 | 3.50 |  | 5 | 1.30 | 2.08 | 1.63 | 0.32 | 4.10 |
| 3-6 | 9 | 2.60 | 2.88 | 1.13 | 1.30 | 4.90 |  | 12 | 2.10 | 2.43 | 1.97 | 0.10 | 5.90 |
| 6-9 | 5 | 3.00 | 2.76 | 1.11 | 1.30 | 4.10 |  | 9 | 2.50 | 2.43 | 0.69 | 1.10 | 3.30 |
| 9-12 | 9 | 1.80 | 2.51 | 1.74 | 0.80 | 5.90 |  | 10 | 2.95 | 2.93 | 1.25 | 1.10 | 5.40 |
| 12-18 | 6 | 3.00 | 2.83 | 1.23 | 1.00 | 4.10 |  | 7 | 2.90 | 2.90 | 1.47 | 1.40 | 5.30 |
| 18-24 | 5 | 0.90 | 1.70 | 1.47 | 0.50 | 3.40 |  | 7 | 1.90 | 2.25 | 1.10 | 1.10 | 3.80 |
| 24-36 | 5 | 1.10 | 1.28 | 0.79 | 0.40 | 2.10 |  | 8 | 3.25 | 3.53 | 1.22 | 2.00 | 5.60 |
| 36-60 | 2 | 1.75 | 1.75 | 1.20 | 0.90 | 2.60 |  | 15 | 2.50 | 2.80 | 1.11 | 1.40 | 5.70 |
|  |  |  |  |  |  |  |  |  |  |  |  |  |  |
|  | **Psychosine** | | | | | | | | | | | | |
| **Age (months)** | **N** | **Median** | **Mean** | **SD** | **Min** | **Max** |  | **N** | **Median** | **Mean** | **SD** | **Min** | **Max** |
| 0-3 | 3 | 13.0 | 10.8 | 5.9 | 4.1 | 15.2 |  | 16 | 10.0 | 11.4 | 8.7 | 1.0 | 29.1 |
| 3-6 | 2 | 54.0 | 54.0 | 0.0 | 54.0 | 54.0 |  | 7 | 3.8 | 4.7 | 2.0 | 2.6 | 8.3 |
| 6-9 | 15 | 26.1 | 27.1 | 10.5 | 13.7 | 49.9 |  | 10 | 1.3 | 1.9 | 1.3 | 0.7 | 4.2 |
| 9-12 | 4 | 5.0 | 9.2 | 9.4 | 3.6 | 23.2 |  | 4 | 1.9 | 1.7 | 0.8 | 0.6 | 2.4 |
| 12-18 | 6 | 4.2 | 4.9 | 3.0 | 1.2 | 8.5 |  | 5 | 1.5 | 1.6 | 0.8 | 0.8 | 2.8 |
| 18-24 | 9 | 5.5 | 6.4 | 4.9 | 1.7 | 17.7 |  | 3 | 3.7 | 4.5 | 2.6 | 2.4 | 7.5 |
| 24-36 | 11 | 4.0 | 4.4 | 2.2 | 1.1 | 7.5 |  | 11 | 3.6 | 4.0 | 2.4 | 1.4 | 9.6 |
| 36-60 | 5 | **3** | 5 | 7 | 2 | 17 |  | 10 | 5 | 5 | 3 | 2 | 12 |
|  |  |  |  |  |  |  |  |  |  |  |  |  |  |
|  | **CSF Protein** | | | | | | | | | | | | |
| **Age (months)** | **N** | **Median** | **Mean** | **SD** | **Min** | **Max** |  | **N** | **Median** | **Mean** | **SD** | **Min** | **Max** |
| 0-3 |  |  |  |  |  |  |  | 13 | 239 | 263 | 154 | 89 | 547 |
| 3-6 | 6 | 261 | 306 | 132 | 222 | 571 |  | 11 | 162 | 172 | 113 | 39 | 404 |
| 6-9 | 29 | 187 | 187 | 95 | 49 | 440 |  | 10 | 167 | 176 | 117 | 37 | 456 |
| 9-12 | 6 | 162 | 163 | 56 | 75 | 233 |  | 4 | 185 | 165 | 67 | 71 | 218 |
| 12-18 | 16 | 221 | 217 | 94 | 64 | 483 |  | 13 | 157 | 150 | 42 | 74 | 216 |
| 18-24 | 11 | 134 | 166 | 96 | 11 | 321 |  | 3 | 101 | 129 | 60 | 89 | 198 |
| 24-36 | 13 | 166 | 157 | 44 | 90 | 212 |  | 9 | 142 | 136 | 51 | 50 | 209 |
| 36-60 | 2 | 280 | 280 | 239 | 111 | 449 |  | 9 | 94 | 98 | 35 | 32 | 156 |
