# Supplementary material for: Correction to “Infantile Krabbe disease (0–12 months), progression, and recommended endpoints for clinical trials”
Source: Ann Clin Transl Neurol. 2025 Jan 9;12(2):455. doi: 10.1002/acn3.52275 (PMC11822787; doi:10.1002/acn3.52275)
Supplement: Supplementary file 1 — Figure S1.. [file ACN3-12-455-s013.pdf]

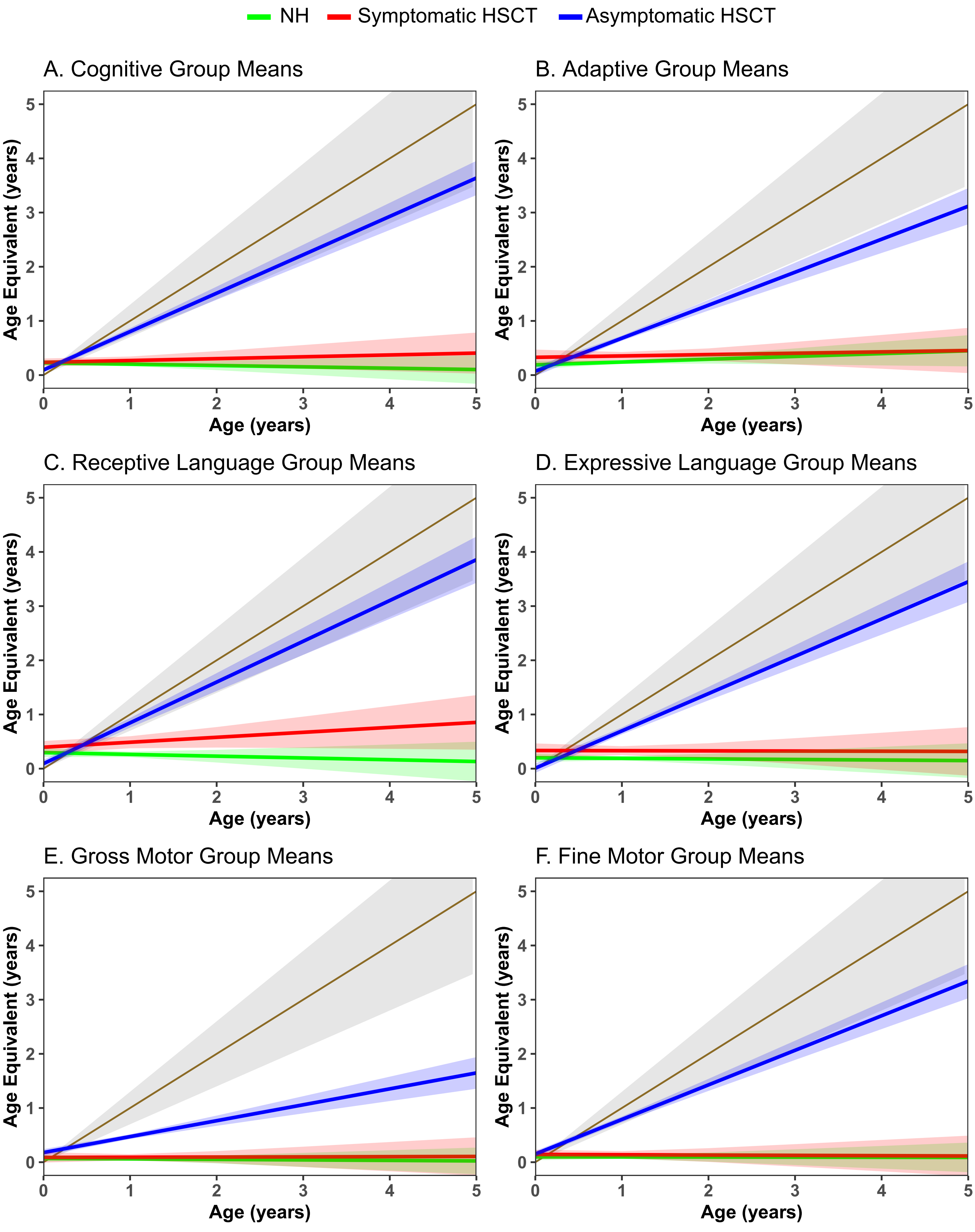

Figure S1. Group mean developmental trajectories. The mean developmental trajectory was estimated for six domains using random effects models. The solid line represents the mean trajectory for the group with the shaded area indicating the 95% confidence interval of the estimates. Natural History patients are in green, Symptomatic HSCT patients are in red, and Asymptomatic HSCT patients are in blue. The grey line and shaded area indicate normal development. The x-axis shows the actual age of the patient, and the y-axis shows the developmental equivalent age. Hematopoietic stem cell transplantation is abbreviated to “HSCT”.
