# Supplementary material for: Correction to “Infantile Krabbe disease (0–12 months), progression, and recommended endpoints for clinical trials”
Source: Ann Clin Transl Neurol. 2025 Jan 9;12(2):455. doi: 10.1002/acn3.52275 (PMC11822787; doi:10.1002/acn3.52275)
Supplement: Supplementary file 2 — Figure S2.. [file ACN3-12-455-s012.pdf]

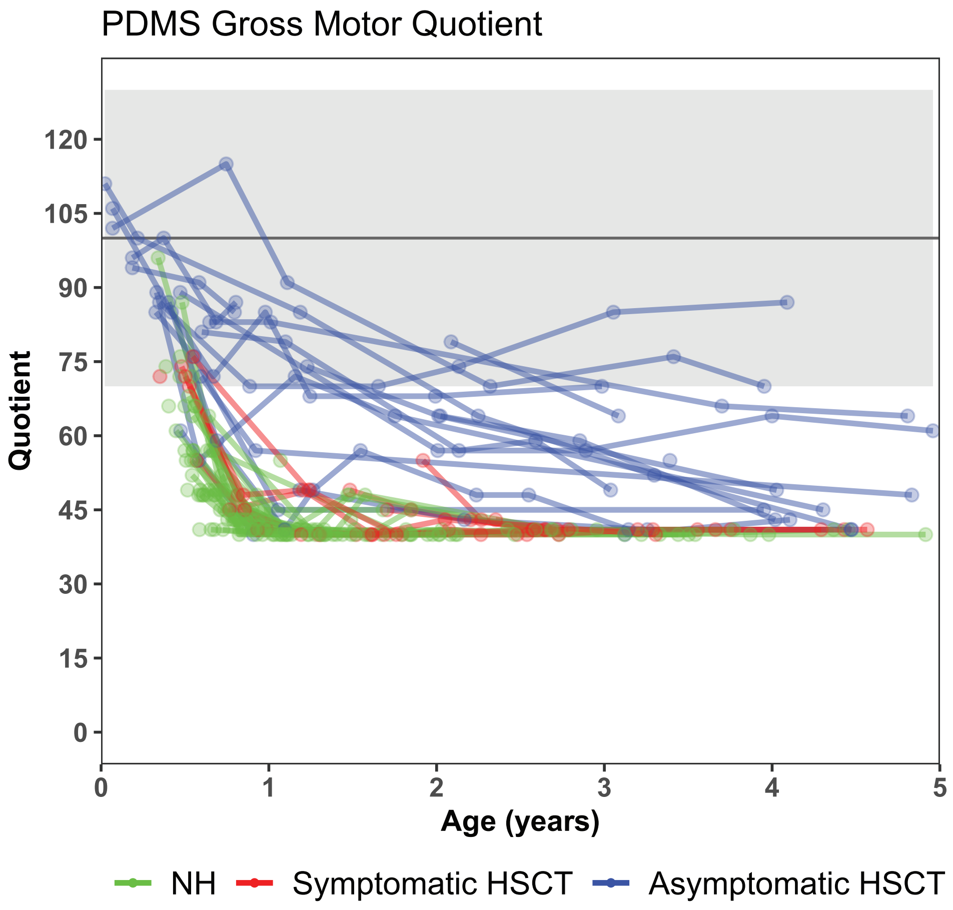

Figure S2. Individual Gross Motor trajectories. Each line represents a patient's Quotient score over time. Natural History patients are in green, Symptomatic HSCT patients are in red, and Asymptomatic HSCT patients are in blue. The grey solid line represents the average typical development over time and the grey shaded area indicates the 95% range of normal development (mean = 100; SD = 15). The x-axis shows the age of the patient and the y-axis shows the Quotient score. Declining trajectories do not necessarily indicate a loss of skills but a failure to acquire skills at a normal rate. To assess skill development, refer to the age equivalent scores in Figure 4E.
