# Supplementary material for: Correction to “Infantile Krabbe disease (0–12 months), progression, and recommended endpoints for clinical trials”
Source: Ann Clin Transl Neurol. 2025 Jan 9;12(2):455. doi: 10.1002/acn3.52275 (PMC11822787; doi:10.1002/acn3.52275)
Supplement: Supplementary file 3 — Figure S3.. [file ACN3-12-455-s001.pdf]

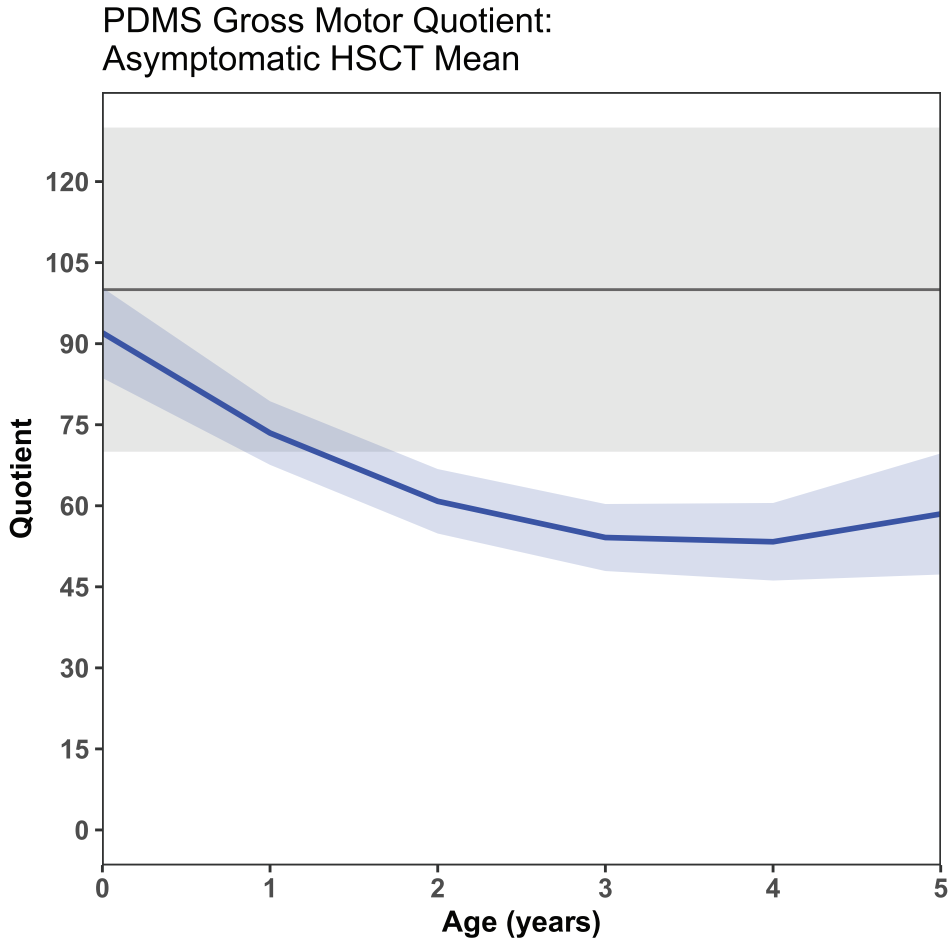

Figure S3. Mean developmental trajectory of Asympt HSCT group for Gross Motor quotient. The mean developmental trajectory was estimated using a random effects model. The blue solid line represents the mean trajectory for the group, with the blue shaded area indicating the 95% confidence interval of the estimates. The grey solid line represents the average normal development over time and grey shaded area indicates 95% distribution of normal development (mean 100; SD = 15). The x-axis indicates the age of the patient and the y-axis indicates the quotient score.
