# Supplementary material for: Correction to “Infantile Krabbe disease (0–12 months), progression, and recommended endpoints for clinical trials”
Source: Ann Clin Transl Neurol. 2025 Jan 9;12(2):455. doi: 10.1002/acn3.52275 (PMC11822787; doi:10.1002/acn3.52275)
Supplement: Supplementary file 4 — Table S1.. [file ACN3-12-455-s003.pdf]

Supplementary Table 1. Genetic variants in natural history patients and patients transplanted symptomatically and asymptomatically.

ID	Group	Allele 1	Allele 2
6	Natural History	p.Arg168Cys_g.30kb deletion	p.Ile546Thr_c.1786+2insT
9	Natural History	p.Y158Lfsx3_p.Q312Q	P.G90R_p.Q312Q
12	Natural History	p.A21P_p.G111D_p.D248N	p.R184C_g.30kb deletion
13	Natural History	p.G36Afsx20	p.G36Afsx20
17	Natural History	30kb deletion	c.592G>A (p.E198K)
18	Natural History	p.Pro148Thr	p.Arg168Cys_g.30Kbdeletion
21	Natural History	p.R168C_p.I546T	g.7.4Kb del_p.I546T
25	Natural History	c.1586C>T (p.Thr529Met)	c.1586C>T (p.Thr529Met)
26	Natural History	24kb Deletion	
27	Natural History	c.1714delinsAGC (p.Asp572Se	c.1714delinsAGC (p.Asp572Serfs*12)
28	Natural History	c.1472delA	c.967G>T (p.G323W)
29	Natural History	p.G284S_p.S434S_p.I546T	p.S434S_p.I546T_p.L634P
37	Natural History	c.195+1G>A	30kb deletion
40	Natural History	p.R168C_30kb deletion	p.R168C_30kb deletion
41	Natural History	c.1541T>C (p.Phe514Ser)	c.909-10A>C
44	Natural History	30kb deletion	30kb deletion
46	Natural History	p.A5P-G9G_p.D232N_p.T513M	p.A5P-G9G_p.D232N_p.T513M
49	Natural History	p.D94D_p.E499K_p.I546T	p.R168C_g.30kb Del
50	Natural History	p.Ile546The_c.1766dupA (p.T	p.Ile546The_c.1766dupA (p.Tyr589fs*1)
52	Natural History	Exon 11-17 deletion	
54	Natural History	p.N279I_p.S434S_p.I546T	p.N279I_p.S434S_p.I546T
55	Natural History	g.30kb deletion	c.1158del10 (p.Met387PhefsX?)
60	Natural History	c.242_243dupAG	c.1700A>C (p.Y567S)
63	Natural History	p.D94D_p.S434S_p.I546T_p.X6	p.I128Lfsx27_p.R168C_p.I546T
65	Natural History	14q31.3-14q32.12	
66	Natural History	p.R168C_g.30kb deletion	p.R168C_g.30kb deletion
69	Natural History	c.387C>G (p.Y129X)	c.1814dupA (p.Y605X)
71	Natural History	Exon 11-17 deletion	c.379C>T (p.R127X)
73	Natural History	c.749T>C (p.I250T)	c.1586C>T (p.T529M)
74	Natural History	p.Arg168Cys_30kb deletion	p.Gly41Ser_p.Ser343Ser_p.Ile546Thr
77	Natural History	Exon 11-17 deletion	Exon 11-17 deletion

82	Natural History	p.R168C_g.30kb del	p.Y319C_p.S434S_p.I546T
83	Natural History	c.1186C>T (p.Arg396Trp)	Exon 11-17 deletion
84	Natural History	p.P239H_p.I546T	p.S434S_p.V320M_p.R515C_p.I546T
86	Natural History	30kb deletion	c.868C>T (p.R290C)
87	Natural History	c.597G>T (p.Arg199Ser)	c.1161+6532_polyA+9kbdel
96	Natural History	Exon 11-17 deletion	Exon 11-17 deletion
97	Natural History	p.R168C_30 kb deletion	c.1637G>A (p.G553R)_c.1786+5 G>A (p.A625T)
101	Natural History	c.281delC (p.Ser94Phefs*5)	c.583-6T>A
103	Natural History	c.908C>T (p.Ser303Phe)	c.1685T>C (p.Ile562Thr)
111	Natural History	Exon 11-17 deletion	c.1793G>A (p.Trp598*)
117	Natural History	p.Ala5Pro_p.Gly9Gly_p.Arg19	p.Ser434Ser_p.Ile546The_p.Leu616Pro
119	Natural History	c.1586C>T (p.T529M)	c.1700A>C (p.Y567S)
121	Natural History	p.R168C_30kb deletion	p.R168C_30kb deletion
126	Natural History	p.W147X_p.S434S_p.I546T	p.W147X_p.S434S_p.I546T
127	Natural History	c.926T>C (p.L309X)	
130	Natural History	p.Ile546Thr_p.Tyr551Ser	c.1110_1119delCATGGTAAC (p.Met371Phefs)
134	Natural History	g.30kb deletion_p.Arg168Cys	g.30kb deletion_p.Arg168Cys
136	Natural History	p.R168C_g.30kb del	p.T513M_p.I546T
137	Natural History	c.1630 G>A (p.Asp544Asn)	c.1630 G>A (p.Asp544Asn)
43	HSCT Symptomatic	p.Arg168Cys_30kb deletion	c.572delA_p.Ile546Thr
62	HSCT Symptomatic	p.R168C_30kb deletion	p.Y319C_p.I546T
95	HSCT Symptomatic	c.388G>A (p.Glu130Lys)	c.388G>A (p.Glu130Lys)
102	HSCT Symptomatic	p.A5P-G9G_p.D232N_p.A290T	p.R168C_g.30kb del
16	HSCT Asymptomatic	del30kb_R168C	c.-335G>A_p.G360Dfs*2#
53	HSCT Asymptomatic	c.622-1G>T	Exon 11-17 deletion
58	HSCT Asymptomatic	c.599C>A (p.Ser200Ter)	c.956A>G (p.Tyr319Cys)
59	HSCT Asymptomatic	p.W162X_p.S434Sp.I546T	p.Y474N_p.S434Sp.I546T
61	HSCT Asymptomatic	c.749T>C (p.I250T)	c.1171_1175het_delCATTCinsA
78	HSCT Asymptomatic	c.658C>T (p.Arg220Ter)	c.956A>G (pTyr319Cys)
79	HSCT Asymptomatic	30kb deletion	30kb deletion
114	HSCT Asymptomatic	c.658C>T (p.Arg220Ter)	c.956A>G (pTyr319Cys)
122	HSCT Asymptomatic	p.R168C_g.30kbdel	p.A209E
