# Supplementary material for: Correction to “Infantile Krabbe disease (0–12 months), progression, and recommended endpoints for clinical trials”
Source: Ann Clin Transl Neurol. 2025 Jan 9;12(2):455. doi: 10.1002/acn3.52275 (PMC11822787; doi:10.1002/acn3.52275)
Supplement: Supplementary file 5 — Table S2.. [file ACN3-12-455-s014.pdf]

Table S2. Neurodevelopmental measures used to assess each developmental domain.

Domain	Scales Used	# Evaluations	% of Evaluations
**Cognitive**		390	100%
	Mullen Scales of Early Learning	339	87%
	Bayley Scales of Infant Development	19	5%
	The Capute Scales	29	7%
	Differential Ability Scales	3	<1%
**Adaptive**		321	100%
	Scales of Independent Behavior-Revised	244	76%
	Vineland III	77	24%
**Receptive and Expressive Language**		349	100%
	Mullen Scales of Early Learning	339	97%
	Preschool Language Scales 3 or 4 ed	6	2%
	Receptive-Expressive Emergent Language Test	4	1%
**Gross Motor**			
	Peabody Developmental Motor Scales (PDMS) - 2	292	100%
	Gross Motor Function Measure -88	237	100%
**Fine Motor**		380	100%
	Mullen Scales of Early Learning	339	89%
	The Capute Scales	39	10%
	Beery-Buktenica Developmental Test of Visual-Motor Integration	2	<1%
