# Supplementary material for: Correction to “Infantile Krabbe disease (0–12 months), progression, and recommended endpoints for clinical trials”
Source: Ann Clin Transl Neurol. 2025 Jan 9;12(2):455. doi: 10.1002/acn3.52275 (PMC11822787; doi:10.1002/acn3.52275)
Supplement: Supplementary file 6 — Table S3.. [file ACN3-12-455-s002.pdf]

Table S3. Racial distribution of study cohort. Six patients chose to not disclose.

Race	Number of patients
White	123
Black	5
Biracial	2
Asian	1
Unknown	6
