# Supplementary material for: Correction to “Infantile Krabbe disease (0–12 months), progression, and recommended endpoints for clinical trials”
Source: Ann Clin Transl Neurol. 2025 Jan 9;12(2):455. doi: 10.1002/acn3.52275 (PMC11822787; doi:10.1002/acn3.52275)
Supplement: Supplementary file 7 — Table S4.. [file ACN3-12-455-s011.pdf]

Table S4. Number of evaluations for each patient by group.

# of Evaluations	Natural History	HSCT Symptomatic	HSCT Asymptomatic
**1**	47	2	2
**2**	22	4	1
**3**	13	3	1
**4**	4	1	1
**5**	5	0	3
**6**	1	3	2
**7**	2	1	3
**8**	1	0	2
**9**	1	2	5
**10**	0	2	2
**11**	0	0	1
**Total**	96	18	23
