# Supplementary material for: Correction to “Infantile Krabbe disease (0–12 months), progression, and recommended endpoints for clinical trials”
Source: Ann Clin Transl Neurol. 2025 Jan 9;12(2):455. doi: 10.1002/acn3.52275 (PMC11822787; doi:10.1002/acn3.52275)
Supplement: Supplementary file 8 — Table S5.. [file ACN3-12-455-s010.pdf]

Supplementary Table 5. Summary of neonatal complications. Multiple concomitant complications included jaundice requiring phototherapy, feeding difficulties or latching problems, respiratory distress, arrythmias or cardiovascular problems, severe hypoglycemia, gastroesophageal reflux and vomiting, and Erb's palsy or clavicle fracture due to complicated delivery.

**Complications**	**Number of patients (%)**
Delivered via C-section	47 (34%)
Neonatal complications	57 (42%)
Multiple concomitant complications	17 (12%)
