# Supplementary material for: Correction to “Infantile Krabbe disease (0–12 months), progression, and recommended endpoints for clinical trials”
Source: Ann Clin Transl Neurol. 2025 Jan 9;12(2):455. doi: 10.1002/acn3.52275 (PMC11822787; doi:10.1002/acn3.52275)
Supplement: Supplementary file 9 — Table S6.. [file ACN3-12-455-s008.pdf]

Table S6. Number and percentage of patients with indicated signs and symptoms by age

	0-2 months		3-5 months		6-8 months		9-11 months		12-17 months		18-23 months		24-35 months		36-60 months	
	N	%	N	%	N	%	N	%	N	%	N	%	N	%	N	%
**Diarrhea**																
NH	0/1	0%	3/14	22%	3/37	8%	1/29	3%	0/35	0%	0/18	0%	3/16	19%	0/10	0%
Sympt	-	-	2/3	68%	0/8	0%	1/4	25%	1/7	14%	0/9	0%	0/8	0%	0/8	0%
Asympt	0/20	0%	0/8	0%	2/10	20%	0/7	0%	1/13	8%	0/9	0%	1/13	8%	0/19	0%
**Constipation**																
NH	0/1	0%	4/15	27%	17/41	41%	19/32	59%	25/38	66%	13/20	65%	11/17	65%	10/11	91%
Sympt	-	-	0/3	0%	3/8	38%	1/4	25%	1/7	14%	0/9	0%	2/8	25%	1/8	13%
Asympt	0/19	0%	0/8	0%	0/10	0%	0/7	0%	0/13	0%	0/9	0%	3/13	23%	3/19	16%
**Feeding Difficulties**																
NH	1/1	100%	13/15	87%	41/43	95%	32/33	97%	38/40	95%	19/19	100%	15/16	94%	10/10	100%
Sympt	-	-	3/6	50%	8/9	89%	4/4	100%	6/7	86%	10/10	100%	7/8	88%	8/8	100%
Asympt	1/20	5%	4/8	50%	4/10	40%	2/7	29%	3/13	23%	3/9	33%	8/13	62%	8/19	42%
**Reflux**																
NH	1/1	100%	13/16	81%	30/37	81%	18/30	60%	17/34	50%	9/19	47%	4/16	25%	7/10	70%
Sympt	-	-	2/6	33%	4/9	44%	2/4	50%	1/7	14%	6/10	60%	2/8	25%	4/8	50%
Asympt	1/19	5%	0/8	0%	0/10	0%	0/7	0%	1/13	8%	1/9	11%	0/13	0%	2/19	11%
**Swallowing Difficulties**																
NH	0/1	0%	11/14	79%	39/42	93%	28/32	88%	37/37	100%	18/18	100%	15/15	100%	10/10	100%
Sympt	-	-	3/6	50%	8/9	89%	4/4	100%	6/7	86%	8/8	100%	8/8	100%	8/8	100%
Asympt	2/20	10%	2/8	25%	2/10	20%	0/6	0%	1/12	8%	0/8	0%	4/11	36%	3/17	18%
**Sialorrhea with need of Suctioning**																
NH	0/1	0%	1/13	8%	10/39	26%	13/32	41%	22/40	55%	17/20	85%	14/17	82%	7/11	65%
Sympt	-		0/4	0%	1/9	11%	1/4	25%	1/7	14%	3/9	33%	4/8	50%	5/8	63%
Asympt	0/20	0%	0/8	0%	0/10	0%	0/7	0%	0/13	0%	0/9	0%	0/13	0%	1/19	5%

	0-2 months		3-5 months		6-8 months		9-11 months		12-17 months		18-23 months		24-35 months		36-60 months	
	**N**	**%**	**N**	**%**	**N**	**%**	**N**	**%**	**N**	**%**	**N**	**%**	**N**	**%**	**N**	**%**
**Head Control**																
NH	0/1	0%	2/14	14%	7/44	16%	7/34	21%	8/41	20%	0/20	0%	1/17	6%	1/11	9%
Sympt	-	-	2/5	40%	3/8	38%	2/4	50%	2/7	29%	5/10	50%	4/8	50%	4/8	50%
Asympt	5/18	28%	3/9	33%	3/10	30%	1/7	15%	4/12	33%	3/9	33%	5/13	38%	9/19	47%
**Sit Unassisted**																
NH	NA	NA	NA	NA	0/41	0%	0/34	0%	0/41	0%	0/20	0%	0/17	0%	0/11	0%
Sympt	NA	NA	NA	NA	0/9	0%	0/4	0%	0/7	0%	0/10	0%	0/8	0%	0/8	0%
Asympt	NA	NA	NA	NA	0/10	0%	2/6	33%	7/13	54%	7/9	78%	12/13	92%	14/18	78%
**Axial Hypotonia**																
NH	1/1	100%	6/14	43%	32/40	80%	32/34	94%	39/41	95%	20/20	100%	16/16	100%	11/11	100%
Sympt	-	-	2/3	67%	6/9	67%	2/4	50%	6/7	86%	9/9	100%	8/8	100%	8/8	100%
Asympt	2/20	10%	4/8	50%	5/10	50%	5/7	71%	9/12	75%	8/9	89%	11/13	85%	15/19	79%
**Appendicular Spasticity**																
NH	1/1	100%	13/13	100%	42/44	95%	31/33	94%	34/41	83%	14/20	70%	10/17	59%	8/11	73%
Sympt	-	-	6/6	100%	7/9	78%	5/5	100%	7/7	100 %	8/8	100%	8/8	100%	8/8	100%
Asympt	3/20	15%	2/8	25%	4/10	40%	3/7	43%	7/11	64%	5/9	56%	9/13	69%	14/19	74%
**Clasped Thumb**																
NH	1/1	100%	12/15	80%	38/44	86%	27/33	82%	32/40	80%	14/20	70%	12/17	71%	3/10	30%
Sympt	-	-	6/6	100%	7/8	88%	4/5	80%	5/7	71%	4/9	44%	4/8	50%	5/8	63%
Asympt	2/20	10%	3/9	33%	3/9	33%	2/7	29%	2/13	16%	1/9	11%	213	15%	4/19	21%
**Absent or Abnormal Deep Tendon Reflexes**																
NH	1/1	100%	11/13	85%	39/42	93%	34/34	100%	41/41	100 %	19/20	95%	17/17	100%	10/10	100%
Sympt	-	-	5/6	83%	8/9	89%	5/5	100%	6/7	86%	9/10	90%	8/8	100%	8/8	100%
Asympt	4/19	21%	3/8	38%	5/10	50%	3/7	43%	10/12	84%	5/8	63%	10/12	83%	16/18	89%

	0-2 months		3-5 months		6-8 months		9-11 months		12-17 months		18-23 months		24-35 months		36-60 months	
	**N**	**%**	**N**	**%**	**N**	**%**	**N**	**%**	**N**	**%**	**N**	**%**	**N**	**%**	**N**	**%**
**Bulging Fontanelle**																
NH	0/1	0%	4/15	27%	10/41	24%	7/33	21%	14/40	35%	9/20	45%	8/17	48%	5/10	50%
Sympt	-	-	1/6	17%	1/9	20%	2/4	50%	2/7	29%	2/10	20%	2/8	25%	0/8	0%
Asympt	4/20	20%	4/7	57%	6/10	60%	4/7	57%	7/13	54%	4/8	50%	1/13	8%	1/18	6%
**Staring Episodes**																
NH	0/1	0%	4/16	25%	17/44	39%	19/34	56%	20/41	49%	17/20	85%	14/17	83%	10/11	90%
Sympt	-	-	1/6	17%	2/9	22%	2/5	40%	1/7	14%	3/11	27%	4/8	50%	3/8	38%
Asympt	0/20	0%	0/10	0%	0/13	0%	1/8	13%	1/13	8%	0/9	0%	1/16	6%	0/20	0%
**Clinical Seizures**																
NH	0/1	0%	1/15	7%	4/40	10%	6/33	18%	10/38	26%	6/19	32%	4/17	24%	4/11	36%
Sympt	-	-	0/1	0%	1/9	11%	1/4	25%	0/7	0%	2/9	22%	1/8	13%	2/8	25%
Asympt	0/19	0%	0/8	0%	0/10	0%	0/7	0%	0/13	0%	1/9	11%	2/13	15%	2/19	11%
**Hip Asymmetry**																
NH	0/1	0%	0/11	0%	6/38	16%	9/33	27%	17/41	41%	8/20	40%	12/17	71%	11/11	100%
Sympt	-	-	0/2	0%	1/9	11%	2/4	50%	2/7	29%	4/10	40%	3/8	38%	3/8	38%
Asympt	0/20	0%	0/8	0%	1/10	10%	0/7	0%	0/13	0%	0/9	0%	1/13	8%	3/17	18%
**Scoliosis**																
NH	0/1	0%	0/13	0%	6/40	15%	8/33	24%	18/39	46%	10/20	50%	10/17	59%	11/11	100%
Sympt	-	-	0/2	0%	0/9	0%	0/4	0%	0/7	0%	2/9	22%	7/8	88%	5/8	63%
Asympt	0/20	0%	0/8	0%	1/10	10%	0/7	0%	0/13	0%	2/9	22%	2/13	15%	3/19	16%

	0-2 months		3-5 months		6-8 months		9-11 months		12-17 months		18-23 months		24-35 months		36-60 months	
	**N**	**%**	**N**	**%**	**N**	**%**	**N**	**%**	**N**	**%**	**N**	**%**	**N**	**%**	**N**	**%**
**Vision and Hearing**																
**Visual Tracking Difficulties**																
NH	0/1	0%	12/16	75%	28/43	65%	20/33	61%	32/41	78%	15/20	75%	15/17	88%	9/11	82%
Sympt	-	-	3/6	50%	4/9	44%	2/5	40%	2/7	29%	3/10	30%	4/8	50%	3/8	38%
Asympt	1/20	5%	2/9	22%	1/10	10%	0/7	0%	0/13	0%	0/9	0%	0/13	0%	0/19	0%
**VEP**																
NH	-	-	2/6	33%	3/17	18%	4/16	25%	7/13	53%	3/8	38%	-	-	2/2	100%
Sympt	-	-	0/1	0%	1/1	100%	-	-	3/5	60%	2/5	40%	3/6	50%	0/5	0%
Asympt	5/8	63%	0/3	0%	0/4	0%	0/5	0%	2/6	33%	1/2	50%	1/3	33%	0/4	0%
**ABR**																
NH	-	-	3/5	60%	17/18	95%	12/13	92%	10/11	91%	6/6	100%	2/3	67%	4/4	100%
Sympt	-	-	1/1	100%	6/6	100%	0/1	0%	6/6	100%	7/7	100%	6/8	75%	2/4	50%
Asympt	8/11	73%	4/4	100%	3/5	60%	2/3	67%	4/6	67%	2/3	67%	4/5	80%	5/6	84%
