# Supplementary material for: Correction to “Infantile Krabbe disease (0–12 months), progression, and recommended endpoints for clinical trials”
Source: Ann Clin Transl Neurol. 2025 Jan 9;12(2):455. doi: 10.1002/acn3.52275 (PMC11822787; doi:10.1002/acn3.52275)
Supplement: Supplementary file 10 — Table S7.. [file ACN3-12-455-s005.pdf]

Table 7. Summary of the percentages of Natural History, Symptomatic HSCT, and Asymptomatic HSCT patients that sat independently by 12 and 24 months of age and walked independently by 24 and 48 months of age.

Sitting independently		
**Group**	**12 months**	**24 months**
Natural History	6%	8%
Symptomatic HSCT	0%	0%
Asymptomatic HSCT	42%	53%
**Walking independently**		
**Group**	**24 months**	**48 months**
Natural History	0%	0%
Symptomatic HSCT	0%	0%
Asymptomatic HSCT	10%	21%
