# Supplementary material for: Correction to “Infantile Krabbe disease (0–12 months), progression, and recommended endpoints for clinical trials”
Source: Ann Clin Transl Neurol. 2025 Jan 9;12(2):455. doi: 10.1002/acn3.52275 (PMC11822787; doi:10.1002/acn3.52275)
Supplement: Supplementary file 11 — Table S8.. [file ACN3-12-455-s015.pdf]

Table S8. Regression model results estimating the developmental trajectories for each neurodevelopmental domain. Random effects models were fit for the age equivalent scores in each of the domains over time. Random intercepts and slopes were estimated except for Adaptive and Receptive Language where only random intercepts were fit. Post-hoc comparisons between the groups at 2.5 and 4.5 years of age were estimated from the regression coefficients. All analyses were age centered at 2.5 years and performed using SAS 9.4 Proc Mixed.

	Cognitive			Adaptive*			Receptive Language*			Expressive Language			Gross Motor			Fine Motor		
**Variable**	**β**	**SE**	**p**	**β**	**SE**	**p**	**β**	**SE**	**p**	**β**	**SE**	**p**	**β**	**SE**	**p**	**β**	**SE**	**p**
Intercept	0.16	0.06	0.005	0.31	0.04	<0.001	0.21	0.05	0.008	0.17	0.07	0.009	0.05	0.05	0.357	0.09	0.06	0.105
Age (years)	-0.02	0.03	0.417	0.05	0.03	0.063	-0.04	0.03	0.441	-0.01	0.04	0.785	-0.01	0.03	0.754	0.00	0.03	0.950
Group			<0.001			<0.001			<0.001			<0.001			<0.001			<0.001
HSCT Asymptomatic	1.70	0.10	<0.001	1.26	0.06	<0.001	1.79	0.08	<0.001	1.55	0.11	<0.001	0.87	0.08	<0.001	1.65	0.09	<0.001
HSCT Symptomatic	0.16	0.11	0.146	0.08	0.06	0.169	0.45	0.09	0.006	0.15	0.11	0.190	0.05	0.09	0.578	0.04	0.10	0.725
Natural History	0.00			0.00			0.00			0.00			0.00			0.00		
Group x Age			<0.001			<0.001			<0.001			<0.001			<0.001			<0.001
HSCT Asymptomatic	0.73	0.05	<0.001	0.54	0.03	<0.001	0.79	0.04	<0.001	0.70	0.06	<0.001	0.30	0.04	<0.001	0.64	0.05	<0.001
HSCT Symptomatic	0.06	0.05	0.254	-0.03	0.05	0.420	0.16	0.04	0.079	0.01	0.07	0.914	0.01	0.05	0.806	0.00	0.05	0.947
Natural History	0.00			0.00			0.00			0.00			0.00			0.00		
**Estimates**	**Diff**	**SE**	**p**	**Diff**	**SE**	**p**	**Diff**	**SE**	**p**	**Diff**	**SE**	**p**	**Diff**	**SE**	**p**	**Diff**	**SE**	**p**
Asympt vs Sympt @ 2.5 years	1.55	0.12	<0.001	1.19	0.07	<0.001	1.34	0.10	<0.001	1.40	0.12	<0.001	0.82	0.10	<0.001	1.62	0.11	<0.001
Asympt vs Sympt @ 4.5 years	2.90	0.22	<0.001	2.31	0.13	<0.001	2.60	0.14	<0.001	2.79	0.26	<0.001	1.40	0.20	<0.001	2.90	0.22	<0.001
Asympt vs NH @ 2.5 years	1.70	0.10	<0.001	1.26	0.06	<0.001	1.79	0.08	<0.001	1.55	0.11	<0.001	0.87	0.08	<0.001	1.65	0.09	<0.001
Asympt vs NH @ 4.5 years	3.17	0.18	<0.001	2.33	0.12	<0.001	3.37	0.13	<0.001	2.95	0.22	<0.001	1.47	0.17	<0.001	2.93	0.18	<0.001
Sympt vs NH @ 2.5 years	0.16	0.11	0.146	0.07	0.07	0.289	0.45	0.09	<0.001	0.15	0.11	0.190	0.05	0.09	0.578	0.04	0.10	0.725
Sympt vs NH @ 4.5 years	0.27	0.21	0.187	0.02	0.14	0.903	0.77	0.15	<0.001	0.17	0.24	0.495	0.08	0.19	0.692	0.03	0.21	0.890

*Model only included random intercepts for random effects.
