# Supplementary material for: Correction to “Infantile Krabbe disease (0–12 months), progression, and recommended endpoints for clinical trials”
Source: Ann Clin Transl Neurol. 2025 Jan 9;12(2):455. doi: 10.1002/acn3.52275 (PMC11822787; doi:10.1002/acn3.52275)
Supplement: Supplementary file 12 — Table S9.. [file ACN3-12-455-s004.pdf]

Table 9. Results from Random Effects Models of PDMS Gross Motor Quotient for Asymptomatic HSCT patients. Model was fit with random intercepts and slopes. Age was centered at 2.5 years. Please note that Symptomatic HSCT and Natural History patients are not included in this analysis as the patients scored below the lowest possible score on the test (45).

**Variable**	**B**	**SE**	**p**
Intercept	56.73	3.05	<0.001
Age (centered at 2.5 years)	-6.71	1.16	<0.000
Age	2.96	0.67	<0.001
**Estimates**	**B**	**SE**	**p**
Asymptomatic HSCT @ 0 years	92.01	4.18	<0.001
Asymptomatic HSCT @ 1 years	73.46	2.95	<0.001
Asymptomatic HSCT @ 2 years	60.83	2.98	<0.001
Asymptomatic HSCT @ 3 years	54.12	3.10	<0.001
Asymptomatic HSCT @ 4 years	53.34	3.59	<0.001
Asymptomatic HSCT @ 5 years	58.48	5.60	<0.001
