# Supplementary material for: Correction to “Infantile Krabbe disease (0–12 months), progression, and recommended endpoints for clinical trials”
Source: Ann Clin Transl Neurol. 2025 Jan 9;12(2):455. doi: 10.1002/acn3.52275 (PMC11822787; doi:10.1002/acn3.52275)
Supplement: Supplementary file 13 — Table S10a.. [file ACN3-12-455-s009.pdf]

Table S10a. GALC, psychosine and CSF protein values for Natural History patients. The values in this table are from patients that have not received HSCT at the time of specimen collection. Age at the time of specimen collection is in the first column, with descriptive statistics of the lab values in the remaining columns. GALC is not expected to change over time; consequently, the value is from the 1st specimen collected from each patient. Psychosine and CSF protein values are calculated longitudinally which means that a single patient can contribute values to multiple age groups.

Galactocerebrosidase

Age (months)	N	Median	Mean	SD	Min	Max
0-36	101	0.1	0.1	0.1	0.0	1.1

Psychosine

Age (months)	N	Median	Mean	SD	Min	Max
0-3	27	11.2	20.0	21.2	1.0	76.9
3-6	10	38.4	36.1	15.4	14.8	54.0
6-9	43	28.3	31.5	16.8	5.9	82.3
9-12	13	16.6	18.4	10.8	5.1	36.4
12-18	18	17.9	19.7	16.0	1.0	60.2
18-24	10	12.0	14.3	9.2	4.0	36.5
24-36	9	9.1	12.4	9.3	2.9	33.0
36-60	8	7.8	8.2	3.8	4.0	13.6

CSF Protein

Age (months)	N	Median	Mean	SD	Min	Max
0-3	13	239	264	154	89	547
3-6	10	250	241	141	39	571
6-9	39	255	236	87	43	387
9-12	13	165	212	157	60	594
12-18	17	208	201	88	51	339
18-24	10	203	191	114	34	375
24-36	8	282	265	133	61	405
36-60	3	135	116	33	77	135
