# Supplementary material for: Correction to “Infantile Krabbe disease (0–12 months), progression, and recommended endpoints for clinical trials”
Source: Ann Clin Transl Neurol. 2025 Jan 9;12(2):455. doi: 10.1002/acn3.52275 (PMC11822787; doi:10.1002/acn3.52275)
Supplement: Supplementary file 14 — Table S10b.. [file ACN3-12-455-s007.pdf]

Table 10b. GALC, psychosine and CSF protein post-HSCT values for patients transplanted symptomatically and asymptomatically. The values in this table were collected after HSCT. Age at the time of specimen collection is in the first column, with descriptive statistics of the lab values in the remaining columns. The values are calculated longitudinally which means that a single patient can contribute values to multiple age groups.

Galactocerebrosidase

	**HSCT Symptomatic**						**HSCT Asymptomatic**					
**Months from HSCT**	**N**	**Median**	**Mean**	**SD**	**Min**	**Max**	**N**	**Median**	**Mean**	**SD**	**Min**	**Max**
0-3	4	3.45	3.25	0.44	2.60	3.50	5	1.30	2.08	1.63	0.32	4.10
3-6	9	2.60	2.88	1.13	1.30	4.90	12	2.10	2.43	1.97	0.10	5.90
6-9	5	3.00	2.76	1.11	1.30	4.10	9	2.50	2.43	0.69	1.10	3.30
9-12	9	1.80	2.51	1.74	0.80	5.90	10	2.95	2.93	1.25	1.10	5.40
12-18	6	3.00	2.83	1.23	1.00	4.10	7	2.90	2.90	1.47	1.40	5.30
18-24	5	0.90	1.70	1.47	0.50	3.40	7	1.90	2.25	1.10	1.10	3.80
24-36	5	1.10	1.28	0.79	0.40	2.10	8	3.25	3.53	1.22	2.00	5.60
36-60	2	1.75	1.75	1.20	0.90	2.60	15	2.50	2.80	1.11	1.40	5.70

Psychosine

**Age (months)**	**N**	**Median**	**Mean**	**SD**	**Min**	**Max**	**N**	**Median**	**Mean**	**SD**	**Min**	**Max**
0-3	3	13.0	10.8	5.9	4.1	15.2	16	10.0	11.4	8.7	1.0	29.1
3-6	2	54.0	54.0	0.0	54.0	54.0	7	3.8	4.7	2.0	2.6	8.3
6-9	15	26.1	27.1	10.5	13.7	49.9	10	1.3	1.9	1.3	0.7	4.2
9-12	4	5.0	9.2	9.4	3.6	23.2	4	1.9	1.7	0.8	0.6	2.4
12-18	6	4.2	4.9	3.0	1.2	8.5	5	1.5	1.6	0.8	0.8	2.8
18-24	9	5.5	6.4	4.9	1.7	17.7	3	3.7	4.5	2.6	2.4	7.5
24-36	11	4.0	4.4	2.2	1.1	7.5	11	3.6	4.0	2.4	1.4	9.6
36-60	5	2.5	5.4	6.6	1.7	17.1	10	4.7	5.5	3.4	1.5	12.2

CSF Protein

**Age (months)**	**N**	**Median**	**Mean**	**SD**	**Min**	**Max**	**N**	**Median**	**Mean**	**SD**	**Min**	**Max**
0-3	-	-	-	-	-	-	13	239	264	154	89	547
3-6	6	261	306	132	222	571	11	162	172	113	39	404
6-9	29	187	187	95	49	440	10	167	176	117	37	456
9-12	6	162	163	56	75	233	4	185	165	67	71	218
12-18	16	221	217	94	64	483	13	157	150	42	74	216
18-24	11	134	166	96	11	321	3	101	129	60	89	198
24-36	13	166	157	44	90	212	9	142	136	51	50	209
36-60	2	280	280	239	111	449	9	94	98	35	32	156
